# Overaccumulation of p53-mediated autophagy protects against betulinic acid-induced apoptotic cell death in colorectal cancer cells

**DOI:** 10.1038/cddis.2017.485

**Published:** 2017-10-05

**Authors:** Sen Wang, Kexin Wang, Chundong Zhang, Wanfeng Zhang, Qian Xu, Yitao Wang, Yulin Zhang, Yi Li, Ying Zhang, Huifang Zhu, Fangzhou Song, Yunlong Lei, Youquan Bu

**Affiliations:** 1 Department of Biochemistry and Molecular Biology, Chongqing Medical University, Chongqing 400016, China; 2 Molecular Medicine and Cancer Research Center, Chongqing Medical University, Chongqing 400016, China; 3 Department of Radiology, Affiliated Hospital, Southwest Medical University, Luzhou, Sichuan 646000, China; 4 Department of Bioinformatics, Chongqing Medical University, Chongqing 400016, China; 5 Department of Anesthesiology, North Sichuan Medical College, Nanchong, Sichuan 637000, China; 6 Department of Basic medicine, Chongqing Medical University, Chongqing, 400016, China

## Abstract

Betulinic acid (BA) exhibits cytotoxic activity against some cancer cells. However, the molecular mechanism of BA against CRC cells was little reported. Here, we proved that BA elicited CRC cells' growth inhibition and apoptosis in a dose-dependent manner. In addition, BA treatment induced autophagy via inhibiting the AKT-MTOR signaling pathway. Inhibition of autophagy by either administration of autophagic inhibitor chloroquine or siRNA-mediated knockdown of ATG5 could augment BA-induced apoptotic cell death as well as inhibition of cell proliferation. Moreover, we found that p53 was firstly activated by short exposure to BA and then was rapidly degraded via the ubiquitin-mediated degradation pathway in both wtp53 and mutp53 CRC cells. Notably, more preferential cytotoxicity of BA was obtained in mutp53 cells (IC50 values: HT29, 125 *μ*M; SW480, 58 *μ*M) rather than wtp53 cells (IC50 values: HCT116, 178 *μ*M). Further experiments demonstrated that siRNA-mediated p53 knockdown attenuated BA-induced autophagy, and forced overexpression of p53 augmented BA-induced autophagy, indicating that p53-enhanced BA-induced autophagy. Moreover, BA enhanced the sensitivity of mutp53 cells to chemotherapy drugs such as 5-FU and ADR by degradation of mutp53. Overall, our study proved that BA could induce CRC cell death by inducing apoptosis and reduce the overaccumulation of BA-induced protective autophagy by degrading wtp53 and mutp53 dependent on the ubiquitin-mediated degradation pathway to achieve killer effect, suggesting that BA might serve as a novel desirable drug for mutp53 cancer therapy.

Colorectal cancer is the third most common cancer and the fourth most common cause of cancer death worldwide.^1, 2^ As alternative treatments, new chemotherapy agents using novel mechanisms to induce CRC cell death are needed, whereas plants are an important source for potential anticancer compounds.^3, 4, 5^ Betulinic acid (BA), a naturally occurring pentacyclic triterpene, has shown anticancer property in several human cancers^6, 7, 8, 9, 10, 11, 12, 13, 14, 15^ and no effect on untransformed cells.^16, 17, 18^ However, the mechanism of BA-induced antitumor effect has been still on debate.^12, 19^ In CRC cells, BA could induce apoptosis through the mitochondrial pathway,^20^ inhibited cell growth of CRC cells by downregulating Sp transcription factors in SW480 and RKO cells,^21^ or by suppressing NF-*κ*B and STAT3 signaling in HT29 cells.^22^ It has also been reported that BA can function as NF-*κ*B activator in a number of other cancer cell lines,^23^ suggesting that the antitumor mechanisms of BA was dependent on tumor type.

Autophagy is a catabolic process that allows cellular macromolecules to be broken down and recycled as metabolic precursors.^24^ Autophagy is usually activated in cancer cells challenged with various stresses.^25, 26, 27, 28, 29^ In response to chemotherapy stress, autophagy probably has four different functions including cytoprotective, cytotoxic, non-protective and cytostatic form.^30, 31, 32, 33, 34, 35^ BA could induce autophagy in multiple myeloma cells and glioblastoma cells,^36^ whereas autophagic flux inhibition promoted apoptosis in human multiple myeloma KM3 cells. Furthermore, BA or BA analog could induce autophagy in HeLa, A549, MCF7, SW480 and HT29 cells, and autophagic salvage counterbalanced BA-induced cell death in HeLa cells.^10, 37^ Although studies observed that BA could induce autophagy, the role of autophagy as a cell death mechanism was not minutely addressed for BA-treated CRC cells.

The tumor suppressor p53 is implicated in a wide range of cellular processes, such as cell-cycle arrest and apoptosis.^38, 39, 40^ p53 binds specifically to DNA and regulates transcription of target genes, such as *P21*, *BIM*, *BAX*, *PUMA* and *NOXA*, to trigger cell death.^41^ However, mutp53 in a large fraction of human cancers could promote evasion of apoptosis, accelerate tumor progression and chemoresistance, which is defined as mutp53 gain-of-function (GOF).^40, 42, 43, 44, 45^ Pharmacological inactivation of mutp53 and effective therapy that targets mutp53 degradation have emerged as a promising strategy to improve cancer therapy.^40, 44, 46^ In addition, p53 can either be a positive or a negative regulator of autophagy.^47, 48^ At basal levels, p53 is recognized as an inhibitor of autophagy through protein–protein interactions in mitochondria.^49, 50^ In response to stress, p53 translocated to the nucleus and promoted autophagy by trans-activating its target genes.^51, 52^ BA triggered an apoptosis pathway different from standard chemotherapeutic drugs because BA-induced apoptosis was independent of p53 in neuroectodermal cancer cells,^53^ melanoma cells and glioblastoma cells.^54^ However, no research was reported regarding whether BA induced CRC cell death independent of p53 status and histotype, and the related detailed mechanism remains unknown.

In this study, we found that BA induced a pro-survival effect of autophagy in CRC cells and p53 can further augment this autophagy. To avoid the overaccumulation of protective autophagy, BA would induce both wtp53 and mutp53 degradation, which makes BA exhibiting preferential cytotoxicity to mutp53 cells.

## Results

### BA inhibits cell proliferation and induces apoptosis in CRC cells

**Figure 1:**
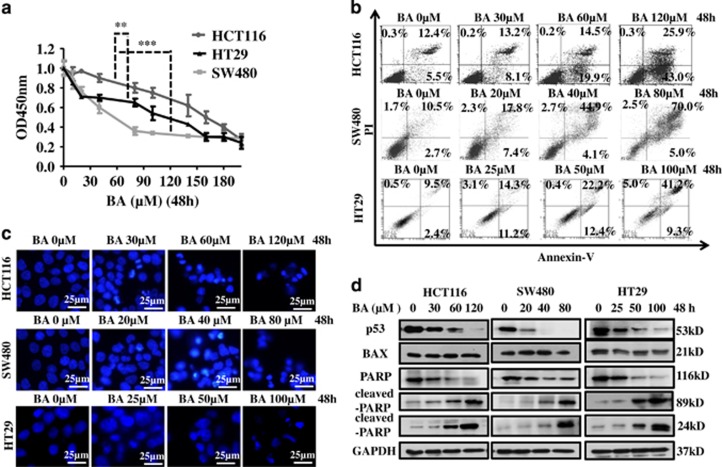
BA inhibits CRC cell proliferation and induces apoptosis. (**a**) Three kinds of CRC cells were seeded into 96-well plates and were treated with the indicated concentrations of BA for 48 h. Cell proliferation assay was performed by CCK8 kit. Data are shown as means±S.D. (*n*=6). Comparisons of IC50 were performed by repeated measures ANOVA and Bonferroni's test using GraphPad Prism software. **P<0.01; ***P<0.0001. (**b**) Three kinds of CRC cells were treated with BA for 48 h at indicated concentrations, and apoptosis rates were analyzed by AV/PI double-staining flow cytometry. (**c**) Three kinds of CRC cells were seeded into 24-well plates and were treated with BA at indicated concentrations for 48 h, and then morphology of the nucleus was observed with the DAPI staining method. (**d**) Three kinds of CRC cells were treated with BA at indicated concentrations for 48 h, and then the apoptosis-related protein was analyzed by immunoblotting. GAPDH was used as the internal control. DMSO was the control group

To determine the anticancer effect of BA in CRC cells, HCT116, SW480 and HT29 cells were treated with different concentrations of BA, and then cell proliferation was assayed with a CCK8 regent kit. We found that administration of BA dose-dependently decreased the proliferation of all three CRC cells. IC50 values of BA against CRC cells were 178 *μ*M (HCT116), 58 *μ*M (SW480) and 125 *μ*M (HT29; Figure 1a). Chemotherapeutic drug-induced cell proliferation inhibition was mainly because of the initiation of apoptosis.^55^ Annexin-V/PI double staining and DAPI staining were used to estimate the effect of BA on apoptosis in CRC cells. The results showed that BA induced apoptosis in a dose-dependent manner (Figures 1b and c). Finally, we further detected the activation of PARP, which is a key factor of apoptosis.^45^ Results showed that BA induced PARP cleavage in a dose-dependent manner (Figure 1d), indicating that BA induced CRC cells' apoptosis. In addition, p53 protein was decreased and its target gene *BAX* was unchanged after BA treatment (Figure 1d), suggesting that p53 may be involved in BA-induced CRC cell death in a special manner.

### BA initiates autophagy in CRC cells

**Figure 2:**
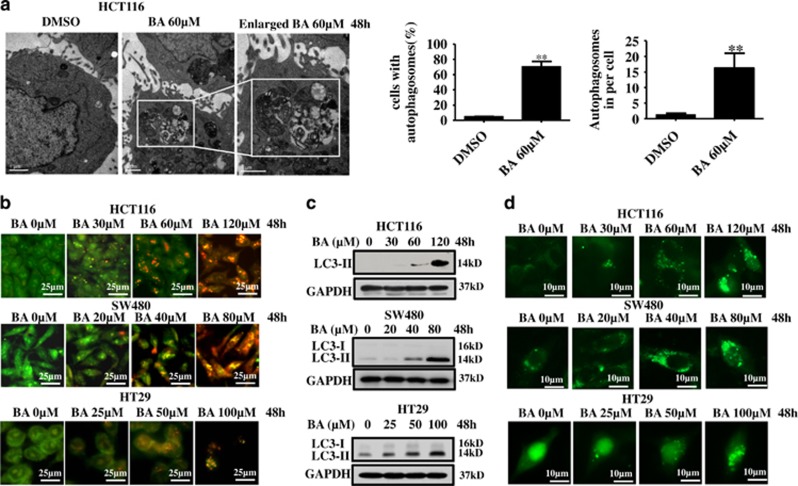
BA induces formation of autophagic vacuoles and AVOs in CRC cells. (**a**) Left: HCT116 cells were treated with BA at 60 *μ*M for 48 h, and then autophagic vacuoles were observed under TEM. DMSO was the control group. Autophagic vacuoles in cells were boxed by the white rectangle and enlarged in the indicated picture. Right: statistical analysis of observation of autophagic vacuoles was described in left. **P<0.01. (**b**) HCT116, SW480 and HT29 cells were treated with BA at indicated concentrations for 48 h, and then acidic autophagic vacuoles were detected by acridine orange staining method. DMSO was the control group. (**c**) HCT116, SW480 and HT29 cells were treated with BA at indicated concentrations for 48 h. Whole-cell lysates were extracted and LC3 protein was detected by immunoblotting. GAPDH was used as internal control. (**d**) HCT116, SW480 and HT29 cells were seeded on the slides in 24-well plates, transfected with GFP-LC3 for 48 h next day and then treated with BA at indicated concentrations for another 48 h. GFP-LC3 puncta was observed under high-magnification fluorescence microscopy

Autophagy has an important role in the drug treatment of cancers.^56^ To investigate whether BA could initiate autophagy in CRC cells, transmission electron microscopy (TEM) was used to monitor autophagy.^24^ The formation of double-membraned autophagic vacuoles was frequently observed in CRC cells treated with BA, but not in control cells (Figure 2a). In addition, we tested the formation of acidic vesicular organelle (AVOs), another major feature of autophagy^57^ in three kinds of CRC cells. Results showed that the numbers of AVOs notably dose-dependently increased in these CRC cells (Figure 2b).

To further confirm whether BA initiates autophagy in CRC cells, the processing of LC3-I to its PE-conjugated LC3-II was measured by immunoblotting. As shown in Figure 2c, BA increased the conversion of LC3-I to LC3-II in a dose-dependent manner, which was further supported by the results that BA treatment increased LC3 punctuates in the cells containing GFP-LC3 plasmids (Figure 2d and Supplementary Figure S1). The distribution of endogenous LC3 in CRC cells was also analyzed by indirect immunofluorescence staining. As shown in Supplementary Figure S2A, more specific LC3 punctuates were found in BA-treated cells compared with the control group. Finally, qRT-PCR was performed to determine the mRNA expression levels of BECLIN1, ATG7, ATG12 and ATG5, which are involved in the formation of autophagosome.^58, 59^ Results showed that BA induced the expression of autophagy-related genes in a dose-dependent manner (Supplementary Figure S2B). These results demonstrated that BA could induce autophagy in CRC cells.

### BA increases the autophagy flux in CRC cells

**Figure 3:**
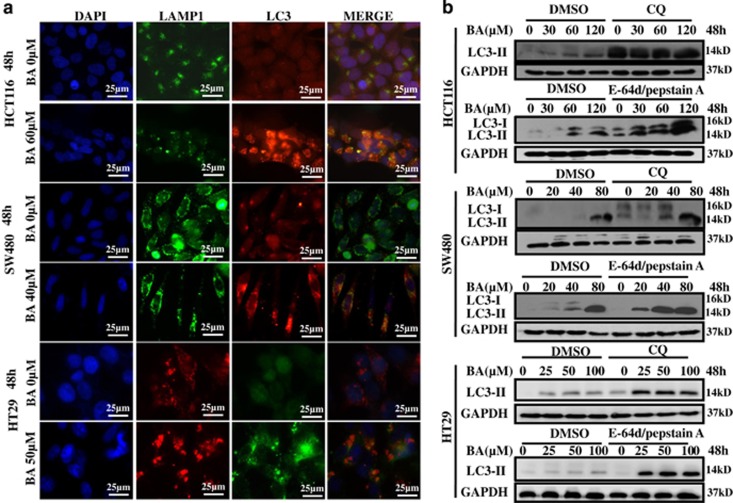
BA induces autophagy flux in CRC cells. (**a**) HCT116, SW480 and HT29 were seeded on slides in 24-well plates and were treated with indicated concentrations of BA for 48 h. Finally, co-location of LAMP1 and LC3 was determined by indirect immunofluorescence. Nucleus was stained with DAPI. LC3 and LAMP1 puncta were observed by high-magnification fluorescence microscopy. (**b**) HCT116, SW480 and HT29 were treated with indicated concentrations of BA for 48 h in the absence or presence of chloroquine (20 *μ*M) or E-64D/Pepstain A (each at 10 *μ*g/ml). LC3 were detected by immunoblotting. GAPDH was used as an internal control. CQ, chloroquine. DMSO was the control group

To determine that BA could also induce autophagy flux in CRC cells, the colocalization of LC3 and LAMP1 was firstly analyzed by indirect immunofluorescence assay, which is often used as a marker for autophagy flux.^7^ The results showed that the colocalization of LC3 and LAMP1 was increased in BA-treated CRC cells compared with the control group (Figure 3a). Another commonly used method for measurement of the autophagy flux is to monitor the conversion of LC3-I to LC3-II in the presence of lysosome inhibitor.^58^ We found that BA co-treatment with E-64 day/pepstain A or chloroquine led to further accumulation of LC3-II in CRC cells (Figure 3b), indicating that BA promoted the autophagy flux in CRC cells. However, interestingly, the mRNA and protein expression of SQSTM1, another marker of the autophagy flux, which is efficiently degraded in the process of autophagy,^60, 61^ were notably increased dose-dependently in the BA treatment group compared with the control group, and SQSTM1 knockdown in HCT116 cells hadn't an obvious influence on BA induced the conversion of LC3-I to LC3-II (Supplementary Figure S3), suggesting that BA-induced autophagy was involved in accumulation of SQSTM1, whereas SQSTM1 did not affect BA-induced autophagy.

### Blockage of autophagy enhances BA-induced proliferation inhibition and apoptosis in CRC cells

**Figure 4:**
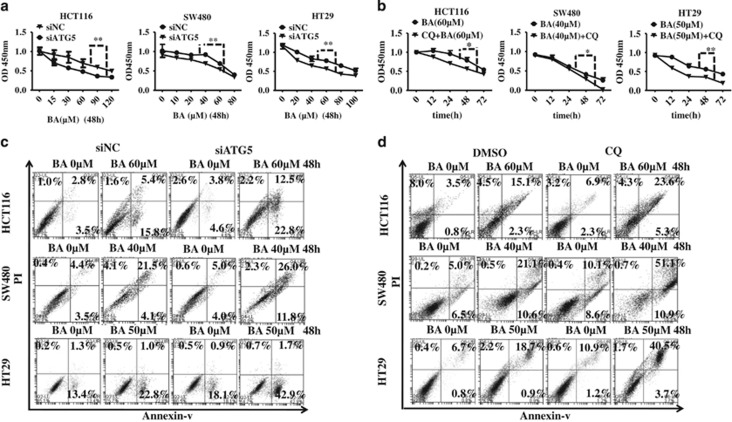
Inhibition of autophagy enhances BA-induced proliferation inhibition and apoptosis in CRC cells. (**a**) Small interference RNA specific to ATG5 and negative control were transfected in HCT116, SW480 and HT29 cells for 24 h and then treated with BA at indicated concentrations for another 48 h. Cell viability was assessed by CCK8 kit. (**b**) HCT116, SW480 and HT29 cells were treated with BA in the presence or absence of CQ (20 *μ*M) for 48 h. Cell viability was analyzed by CCK8 kit. Data are means±S.D. (*n*=5 or 6, Student's *t*-test). *P<0.05, **P<0.01. (**c**) Small interference ATG5 and negative control were transfected in HCT116, SW480 and HT29 cells for 24 h and then treated with the indicated BA for 48 h. Cell apoptosis rates were detected by AV/PI double-staining flow cytometry. DMSO was the control group. (**d**) HCT116, SW480 and HT29 cells were treated with BA at indicated concentrations in the presence or absence of CQ (20 *μ*M) for 48 h. Cell apoptosis rates were detected by AV/PI double-staining flow cytometry. DMSO was the control group

To determine the role of autophagy on BA-induced antitumor effect in CRC cells, we inhibited autophagy by autophagy key gene silencing method or administration of autophagy inhibitors. Results showed gene silencing of autophagy key gene *ATG5* or administration of autophagy inhibitors chloroquine notably enhanced BA-induced proliferation inhibition and apoptosis in CRC cells (Figure 4 and Supplementary Figures S4 and S5). Consistently, the cleavage of pro-apoptosis protein PARP was also enhanced in ATG5 silencing or CQ treatment in contrast to the control (Supplementary Figure S5). Besides that, another autophagy key gene *BECLIN1* silencing and co-treatment with another autophagy inhibitor 3-MA, an inhibitor blocking the initial step of autophagy,^62^ also successfully inhibited BA-induced conversion of LC3-I to LC3-II and promoted BA-induced cleavage of pro-apoptosis protein PARP (Supplementary Figure S5). Taken together, these results demonstrate that autophagy has a protective role in BA-treated CRC cells.

### The AKT-MTOR signaling pathway was involved in BA-induced autophagy

**Figure 5:**
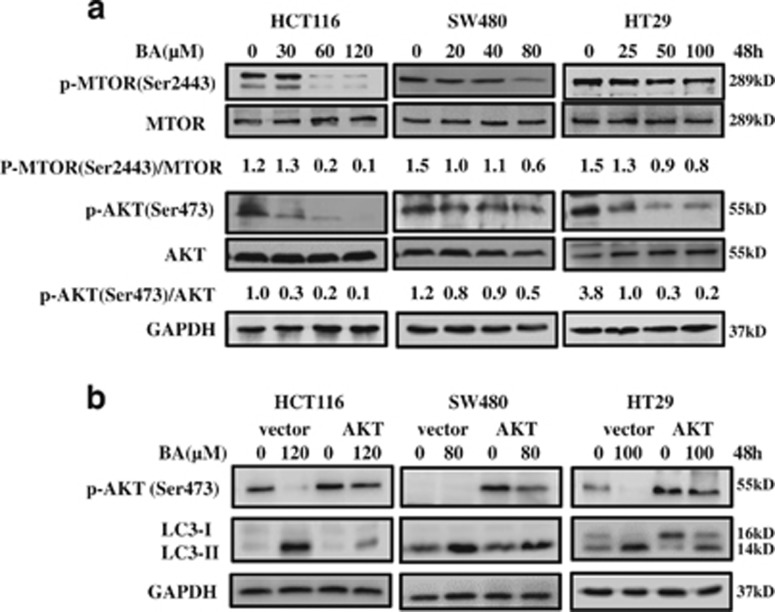
BA induces autophagy by inhibiting the AKT-MTOR signaling pathway. (**a**) HCT116, SW480 and HT29 cells were treated with indicated concentration of BA for 48 h, and then total MTOR, total AKT, phosphorylation of MTOR (P-MTOR) and phosphorylation of AKT (P-AKT) were analyzed by immunoblotting. The ratios of P-MTOR/MTOR and P-AKT/AKT were calculated with Image J. (**b**) Myr-AKT-delta vectors and control DNA were transfected into HCT116, SW480 and HT29 cells for 48 h, and then the transfected cells were treated with indicated concentrations of BA for 48 h. P-AKT and LC3 were detected by immunoblotting. GAPDH was used as an internal control. DMSO was the control group

The AKT-MTOR signaling is reviewed as a key negative regulator of autophagy.^63^ Therefore, we verified whether BA-induced autophagy is also the case in CRC cells. As shown in Figure 5a, BA treatment inhibited phosphorylation of both AKT (S473) and MTOR (S2448) in HCT116, SW480 and HT29 cells. In addition, overexpression of continuous activation Myr-AKT-delta vector decreased BA-induced conversion of LC3 contrast with control in three kinds of CRC cells (Figure 5b), suggesting that BA could induce autophagy by inhibiting AKT-MTOR signaling in CRC cells.

### BA first activated and then rapidly degraded p53 by the ubiquitin–proteasome pathway in mutp53 and wtp53 harboring CRC cells

**Figure 6:**
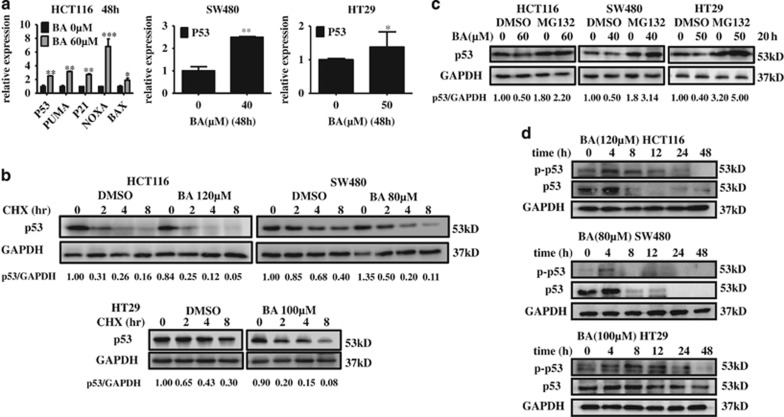
BA first activated and then rapidly degraded p53 by the ubiquitin–proteasome pathway in mutp53 and wtp53 harboring CRC cells. (**a**) HCT116, SW480 and HT29 cells were treated with BA at the indicated concentrations for 48 h, and then mRNA levels of P53, PUMA, P21, NOXA and BAX in HCT116 cells and mRNA levels of P53 in SW480 and HT29 were determined by qRT-PCR. (**b**) HCT116, SW480 and HT29 cells were treated with CHX (100 *μ*g/ml) and/or BA at the indicated concentrations for the indicated time, and then the p53 level was detected by immunoblotting. GAPDH was used as an internal control. The ratio between GAPDH and p53 quantification was determined by Image Lab. (**c**) HCT116, SW480 and HT29 cells were treated with BA and/or MG132 (20 *μ*M) for 20 h, an inhibitor of proteasome, and then the p53 protein level was detected by immunoblotting. GAPDH was used as an internal control. The ratio between GAPDH and p53 quantification was determined by Image Lab. DMSO was the control group. (**d**) HCT116, SW480 and HT29 cells were treated with BA at the indicated concentrations at times of 0, 4, 8, 16, 24 and 48 h. Then, p53 and phosphorylation p53 at S15 were determined by immunoblotting. GAPDH was internal control. DMSO was the control group

To analyze why and how BA affects p53 expression, three kinds of CRC cells harboring either mutant p53 (SW480 and HT29) or wild-type p53 (HCT116) were analyzed by immunoblotting. As shown in Figure 1d, BA strongly reduced the levels of mutp53 and wtp53 protein, but increased their mRNA levels and the mRNA expression of wild-type p53-targeted genes (such as *NOXA*, *PUMA*, *P21* and *BAX;* Figure 6a), suggesting that BA can enhance p53 degradation. To investigate the mechanisms of BA-induced p53 degradation, we next performed cycloheximide chase experiment. As shown in Figure 6b, BA markedly decreased the half-life of both wtp53 and mutp53 proteins. However, proteasome inhibition by MG132 rescued BA-induced degradation of wtp53 and mutp53 (Figure 6c). In addition, autophagy inhibition did not affect BA-induced p53 protein degradation and MDM2 protein was also decreased by BA (Supplementary Figures S5A and B), indicating that BA-induced p53 protein degradation is not dependent on autophagy regulation and its negative regulator, the E3 ubiquitin ligase MDM2. Taken together, our results indicated that BA degraded both wild-type p53 and mutant p53 protein by ubiquitin–proteasome pathway independent of MDM2. Owing to that BA can induce transcription of p53 and activation of p53 target gene, p53 may be activated and involved in regulating CRC apoptosis in the early stage of BA treatment. To this end, we examined a short kinetic of p53 quantity after BA treatment for 0, 4, 8, 12, 24 and 48 h. Results showed that BA induced p53 expression and activation till 12 h in HCT116 and HT29 cells and till 4 h in SW480 cells, and then rapidly degraded p53 in three CRC cells (Figure 6d; Etoposide was used as a positive control to activate p53 in CRC cells (Supplementary Figure S6)), suggesting that short exposure to BA firstly activates p53 and then rapidly degrades p53 by the ubiquitin–proteasome pathway.

**Figure 7:**
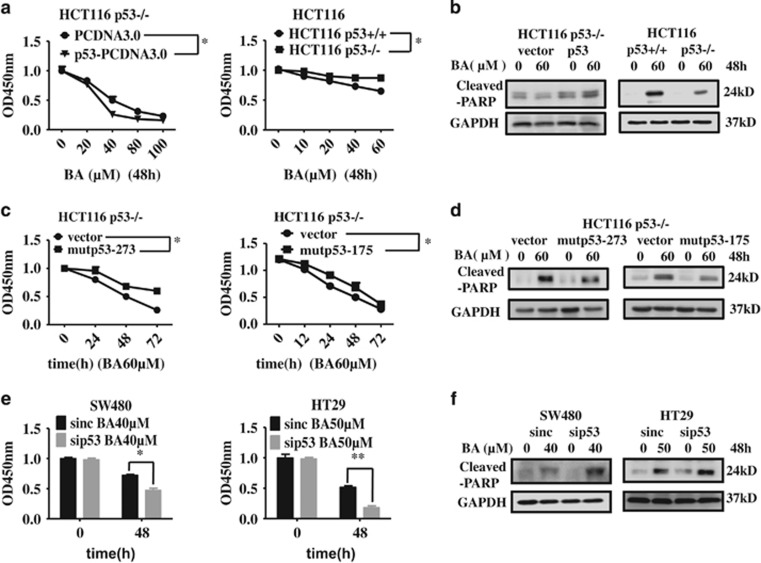
p53 was involved in BA-induced cell proliferation inhibition and apoptosis in CRC cells. (**a** and **b**) Left, HCT116 p53−/− cells were transfected with DNA-p53 or control vector for 48 h, and were incubated with the indicated concentrations of BA or control (DMSO) for another 48 h. Cell viability was detected by CCK8 kit in 96-well plates and whole-cell lysates from six-well plates were collected to analyze the indicated proteins by immunoblotting. Right, HCT116 p53+/+ and p53−/− cells were treated with the indicated concentrations of BA or control (DMSO) for 48 h. Cell viability was detected by CCK8 kit in 96-well plates and whole-cell lysates from six-well plates were collected to analyze the indicated proteins by immunoblotting. (**c** and **d**) HCT116 p53−/− cells were transfected with DNA-mutp53-R273H, DNA-mutp53-R175H or control vector for 48 h, and were incubated with 60 *μ*M of BA or control (DMSO) for another 48 h. Cell viability was detected by CCK8 kit in 96-well plates and whole-cell lysates were collected to analyze the indicated proteins by immunoblot. (**e** and **f**) SW480 and HT29 cells were transfected with siRNA specific to p53 and negative control for 24 h, and then treated with BA at 40 or 50*μ*M for another 48 h. Cell proliferation was measured by CCK8 kit in 96-well plates and whole-cell lysates were collected to analyze the indicated proteins by immunoblotting. Data are shown as means±S.D. (Student's *t-*test). *P<0.05, **P<0.01. DMSO was the control group

**Figure 8:**
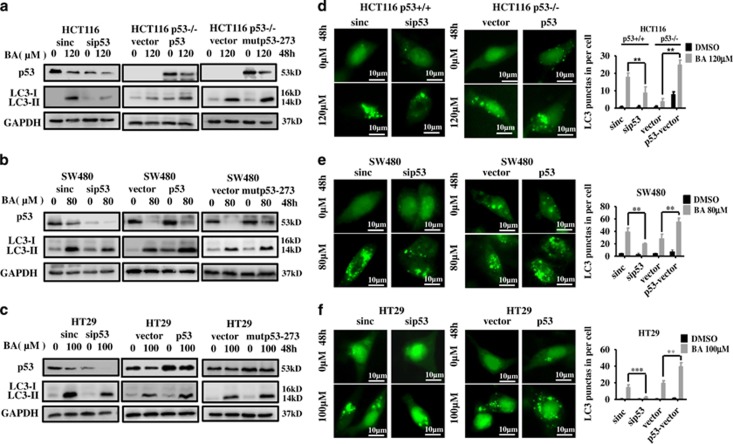
p53 can augment BA-induced autophagy. (**a**–**c**) Left, HCT116, SW480 and HT29 cells were transfected with siRNA targeting p53 and negative control for 24 h, and then were incubated with or without BA at the indicated concentrations for another 48 h. The protein levels of p53, LC3 and internal control (GAPDH) were analyzed by immunoblot. (**a**–**c**) Middle, HCT116 p53 −/−, SW480 and HT29 cells were transfected with DNA-p53 and control vector for 48 h and then incubated with or without the indicated concentrations of BA for another 48 h. The protein levels of p53, LC3 and internal control (GAPDH) were analyzed by immunoblotting. (**a**–**c**) Right, HCT116 p53−/−, SW480 and HT29 cells were transfected with DNA-mutp53-R273H and control vector for 48 h and then were incubated with or without the indicated concentrations of BA for another 48 h. The protein levels of p53, LC3 and internal control (GAPDH) were analyzed by immunoblotting. (**d**–**f**) Left, HCT116, SW480 and HT29 cells were transfected with siRNA targeting p53 and negative control for 24 h, and then were transfected with GFP-LC3 vector for another 24 h, and finally incubated with or without the indicated concentrations of BA for 48 h. GFP-LC3 puncta was observed with a high-magnification fluorescence. DMSO was control. (**d–f**) Right, HCT116 p53−/−, SW480 and HT29 cells were co-transfected with DNA -p53 and GPF-LC3 or control vector and GFP-LC3 for 48 h, and then were treated with the indicated concentrations of BA for another 48 h. GFP-LC3 puncta was observed with a high-magnification fluorescence. DMSO was the control group

Finally, we measured the influence of p53 on BA-induced apoptosis by gain and lose of p53 or mutp53. Interestingly, knockout of p53 unexpectedly restrained BA-induced proliferation inhibition and apoptosis in HCT116 cells (Figures 7a, b and 8a middle), suggesting that early stage of BA-induced p53 may have effects on apoptosis in HCT116 cells. In addition, mutp53-R273H or mutp53-R175H (another important hotspot mutation that usually occurred in cancer^43^) overexpression decreased BA-induced proliferation inhibition and apoptosis in HCT116 p53−/− cells (Figures 7c and d; Supplementary Figure S7A; Figure 8a, right), whereas p53 silencing augmented BA-induced proliferation inhibition and apoptosis in SW480 and HT29 cells with mutation p53 (Figures 7e and f; Supplementary Figure S7B; Figures 8b and c, left), indicating that mutp53 expression enhanced CRC cells' resistance to BA. Interestingly, BA still effectively induced the expression of p53 target genes (*PUMA*, *P21*, *NOXA* and *BIM*) after inhibition of p53 in three CRC cells (Supplementary Figure S8). Considering p53 mutation in SW480 and HT29 cells, other proteins may have an important role in BA-induced cell proliferation inhibition and apoptosis in addition to p53.

### p53 augmented BA-induced protective autophagy and autophagy flux

As BA treatment downregulated p53, we investigated whether loss of p53 involved in BA-induced autophagy. Interestingly, p53 knockdown attenuated BA-induced LC3-II expression (Figures 8a–c, left) and inhibited BA-induced accumulation of GFP-LC3 puncta in CRC cells (Figures 8d–f, left). p53 overexpression increased BA-induced LC3-II expression (Figures 8a–c, middle) and GFP-LC3 puncta in HCT116 p53−/−, SW480 and HT29 cells (Figures 8d–f, right). In addition, BA-induced LC3-II protein expression in HCT116 p53+/+ was more than that in BA-treated HCT116 p53−/− cells (Supplementary Figure S9A) and endogenic LC3 puncta in p53-knockdown HCT116 and HT29 cells also decreased compared with the control group (Supplementary Figure S9B). To determine the role of p53 in BA-induced autophagy flux in CRC cells, a tandem monomeric RFP-GFP-tagged LC3 was used to measure the rate of delivery of autophagosomes to lysosomes. The results showed that BA increased the numbers of both autophagosomes and autolysosomes in CRC cells, which was heightened by p53 overexpression or was restored by silencing p53 (Supplementary Figure S10–S12). These results were confirmed by the evidence that p53 overexpression resulted in further increased BA-induced LC3-II conversion, while p53 silencing inhibited BA-induced LC3-II conversion in the presence or absence of CQ (Supplementary Figure S13). These results demonstrated that p53 could enhance BA-induced autophagy and autophagy flux.

Notably, low dose of ectopic p53 overexpression enhanced BA-induced protective autophagy, whereas high dose of p53 overexpression would reduce BA-induced LC3 conversion (Supplementary Figure S14). In addition, it is known that several miRNAs (e.g., miR34a, miR-218 and miR-502) were involved in p53-mediated cell cycle regulation, autophagy and apoptosis in CRC cells.^64, 65, 66^ Interestingly, we found that BA treatment can induce miR-218 expression in three CRC cells, as well as significantly enhance miR-502 expression in HCT116 and HT29 cells and slightly accumulate miR-502 in SW480 cells (Supplementary Figure S15). These lines of evidence suggested that p53-associated non-coding miRNAs were involved in regulating BA-induced apoptosis and autophagy.

### BA improves chemotherapeutic response in mutp53 CRC cells by degrading mutp53

**Figure 9:**
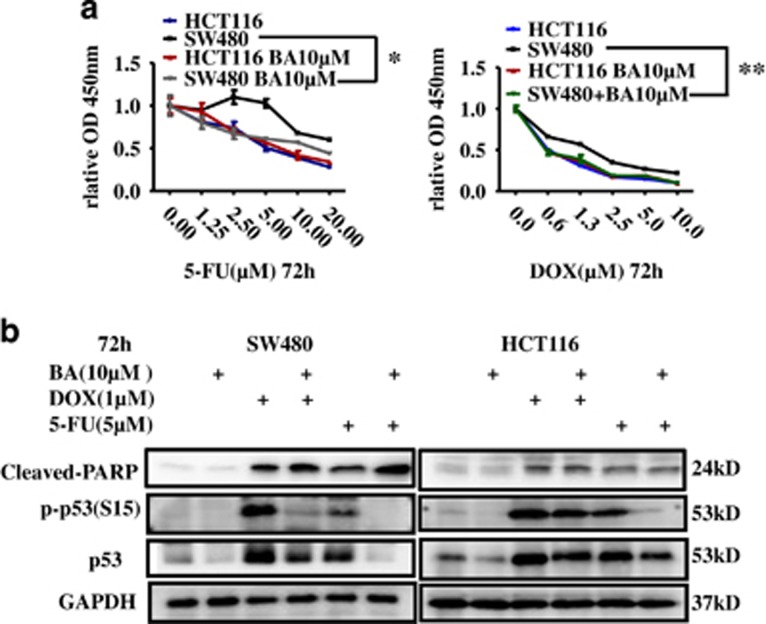
BA improves chemotherapeutic response in mutp53 CRC cells. (**a**) Left, HCT116 and SW480 were treated with 5-FU alone or 5-FU in combination with BA (10 *μ*M) for 72 h. Then, cell viability was analyzed by CCK8 kit. Right, HCT116 and SW480 were treated with ADR alone or ADR in combination with BA (10 *μ*M) for 72 h. Then, cell viability was analyzed by CCK8 kit. Data are shown as means±S.D. (*n*=6, Student's *t-*test). *P<0.05, **P<0.01. (**b**) SW480 and HCT116 cells were treated with 5-FU or ADR at the indicated concentrations alone or in combination of BA (10 *μ*M) for 72 h, respectively. Whole-cell lysates were collected and the indicated proteins were analyzed by immunoblotting. GAPDH was used as internal control. DMSO was the control group

Many studies proved that high expression levels of mutp53 proteins in cancer cells have acquired GOF that actively contribute to cancer development progression, and mutp53 depletion in human cancer cells caused chemosensitization toward an array of conventional genotoxic drugs.^44, 45, 67^ Both BA-induced mutp53 degradation and its associated inhibition of protective autophagy can improve sensitivity of mutp53 cancer cells in response to drug treatment. Our results also found that mutp53 cells (SW480 and HT29) were much more sensitive to BA than wild-type p53 cells (HCT116; Figures 1a and b). Therefore, we asked whether pharmacological degradation of mutp53 by BA could also mediate chemosensitization in response to conventional genotoxic drugs such as 5-FU and Doxorubicin. Results showed that both DOX and 5-Fu induced p53 expression and phosphorylation at Ser15, which could be declined by BA treatment at 10 *μ*M. Moreover, as expected, 10 *μ*M BA, which alone did not induce apparent cell death, synergizing with 5-FU or Doxorubicin caused more cell deaths in mutp53 cells (SW480 cells) in contrast with wtp53 cells (HCT116) (Figures 9a and b). These results suggested that BA could improve other chemotherapeutic drugs partly dependent on degraded mutated p53 in CRC cells.

## Discussion

BA is a bioactive pentacyclic lupine-type triterpenoid natural compound that exhibits anticancer properties.^4, 6, 8, 9^ Here, we reported that BA exerted its anticancer effect by triggering cell proliferation inhibition and apoptosis in CRC cells. In addition, BA induced a protective autophagy by inhibiting the AKT-MTOR signaling pathway, and p53 can further augment this autophagy in CRC cells. To avoid the overaccumulation of protective autophagy, BA would degrade both wtp53 and mutp53, which makes preferential cytotoxicity of BA to mutp53 cells.

Autophagy is a tightly regulated catabolic process of cellular self-digestion by which cellular components are targeted to lysosomes for their degradation.^25^ In response to cancer therapy, autophagy has four faces of function, which are characterized as cytoprotective, cytotoxic, cytostatic and non-protective.^30^ It was demonstrated that BA blocked autophagic flux in KM3 cells and the inhibition of autophagic flux contributed to BA-mediated apoptosis of KM3 cells.^7^ In addition, autophagy was also activated as a response to the mitochondria damage inflicted by BA, which served as a rescue pathway in HeLa, MCF7, A549 and SW480 cells.^10^ However, B10, a new glycosylated derivative of BA, induced autophagy and abrogated the autophagic flux. By concomitant induction of autophagy and inhibition of the autophagic flux, B10 turns autophagy into a cell death mechanism.^36^ Moreover, another research showed that BA decreased phosphorylation of AKT and degraded EGFR in company with induction of autophagy in bladder cancer, which also resulted in autophagic cell death.^68^ In our study, we found BA-induced autophagy by reducing phosphorylation of AKT and MTOR in CRC cells, and autophagy inhibition caused more cell death, indicating that BA-induced autophagy by AKT-MTOR signaling has a cytoprotective effect in CRC cells.

p53 has been linked with regulation of autophagy.^47, 51, 69^ However, the exact nature of this link remains seemingly controversial. It has been reported that nuclear p53 facilitates autophagy by trans-activating its target genes, whereas cytoplasmic p53 mainly inhibits autophagy by transcription-independent mechanisms.^52^ However, these previous observations exclusively reached by p53 or mutp53 overexpression assays are not confirmed by our investigation. Indeed, we revealed that p53 overexpression enhanced BA-induced autophagy, whereas p53 depletion had the contrasting effect, which indicated p53 augmented BA-induced protective autophagy. Besides that, it is also worth mentioning another finding in our study that low overexpression of p53 increased BA-induced autophagy, whereas high overexpression of p53 decreased BA-induced autophagy (Supplementary Figure S14). It may be explained that BA cannot effectively regulate the function of p53 when exogenetic p53 overexpression exceeds a certain amount and p53 may inhibit autophagy by other signaling processes, which is not directly dependent on BA treatment. Autophagy can be regulated by p53-associated non-coding RNAs (for example, miR34a, miR-218 and miR-502).^65, 70, 71, 72^ In this study, we found that BA treatment induced miR-218 expression in three CRC cells, whereas it significantly enhanced miR-502 expression in HCT116 and HT29 cells and slightly augmented it in SW480 cells (Supplementary Figure S15). miR-218 has been observed to induce CRC cell apoptosis by inducing p53 expression^64^ and AKT-mTOR inhibition, which is a major pathway to regulate autophagy.^73^ However, miR-502, which could inhibit autophagy in CRC cells, had potential p53-binding sites in their putative promoter regions and was regulated by p53 via a negative feedback regulatory mechanism.^65, 74^ Therefore, it is possible that BA upregulates miR-218 to induce p53 within a short-time treatment and p53 degradation may increase miR-502, which will inhibit protective autophagy overaccumulation.

As we all know, wtp53 as a tumor suppressor protein has been implicated in apoptosis.^69^ It was reported that BA triggered apoptosis without activation of p53 in neuroectodermal and breast cancers.^53, 75^ Thus, it undoubtedly prompted us to question why and how BA treatment affects p53 expression in CRC cells. We found that BA firstly induced the expression and activation of p53 and then rapidly degraded p53 in both wtp53 and mup53 CRC cells (Figure 6d). In addition, BA further promoted protective autophagy and apoptosis in HCT116 p53+/+ cells compared with HCT116 p53−/− cells (Figure 7b and 8a; Figure 8a, middle and 8D, right; Supplementary Figure S9A), which maybe because that BA activates p53 till 12 h to induce apoptosis in HCT116 p53+/+ cells and then rapidly degraded p53 to inhibit protective autophagy, but the early stage of BA-induced p53 expression has a stronger effect on apoptosis than BA-induced and p53-enhanced protective autophagy. Moreover, mutp53 transfection enhanced BA-induced autophagy and inhibited the sensitivity of HCT116 p53−/− to BA (Figures 7c and d), whereas mutp53 silencing in mutp53-harbored SW480 and HT29 cells inhibited BA-induced protective autophagy and augmented BA-induced apoptosis (Figure 7f and Supplementary Figure S7B), suggesting that this mutp53-promoted and BA-induced autophagy have a role in mutp53-induced BA resistance in CRC cells. In addition, BA upregulated p53 target gene in the presence or absence of p53 in mutp53 and wtp53 CRC cells (Figure 6a and Supplementary Figure S8), suggesting that alternative pathways apart from p53 are involved in BA-induced upregulation of p53 target gene.

There is growing evidence that mutp53 is a clinically relevant target for intervention.^76^ The hyperstability of mutp53 (including p53R273H mutants in HT29 and p53R273H/P309S mutants in SW480) is the basis for its GOF and dominant negativity that promotes malignancy and chemoresistance.^39, 40, 45, 67^ mutp53 is more resistant to proteasome-dependent degradation compared with wtp53, but the exact identity of the pathway is still on debate.^38, 40^ Thus, preventing mutp53 accumulation provides an important chemopreventive and chemotherapeutic strategy. Our study showed that BA preferentially degraded mutp53 through the ubiquitin–proteasome pathway (Figure 6c), indicating that BA treatment may overcome some forms of drug resistance in mutp53 cancers. It has been reported that cytotoxic drugs are given in combinations to enhance their antitumor effectiveness during polychemotherapy.^77^ To this end, we further investigated the application of BA combination in clinical therapy and found that low concentrations of BA combination with chemotherapy drugs such as 5-FU and DOX further caused cell killing than chemotherapy drug treatment alone in mutp53 cancer cells by partly inducing mutp53 degradation (Figures 9a and b), indicating that BA may have an increased therapeutic effect on mutp53-harboring cancers in clinical application.

**Figure 10:**
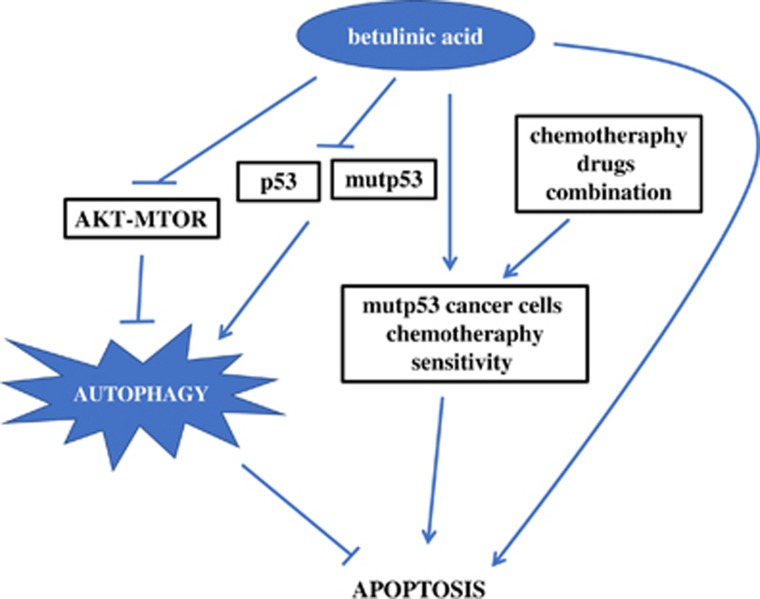
Model of the molecular mechanism unraveled in the presented study. BA induced a protective autophagy by inhibiting AKT-MTOR signaling and degraded p53 to attenuate this protective autophagy overaccumulation, which makes BA acting as one attractive drug in combining with other chemotherapy agents to treat mutp53 CRC cells

Overall, this study highlights that BA induced a protective autophagy by inhibiting AKT-MTOR signaling. In addition, BA firstly activated and then rapidly degraded p53 to attenuate this protective autophagy, which makes BA act as one attractive drug in combination with other chemotherapy agents to treat mutp53 CRC cells (Figure 10). Our findings revealed that BA induced CRC cell death and BA reduced the incidence of mutp53 cancer chemotherapy resistance by degrading mutp53 may have important implications if used for cancer therapy.

## Materials and methods

### Cells and cell culture

Human colorectal cancer HCT116 (wtp53) cells, SW480 (p53R273H/P309S) cells and HT29 (p53R273H) cells were purchased from Shanghai Cell Bank in China. Cells were maintained in DMEM (HyClone, Logan, UT, USA) supplemented with 10% heat-inactivated fetal bovine serum (GIBCO, Carlsbad, CA, USA), penicillin and streptomycin (Hyclone). All cells were maintained in a humidified 5% CO_2_ atmosphere at 37 °C.

### Reagents and antibodies

BA (Meilunbio, Dalian, China; MB5971), Chloroquine (Sigma, St. Louis, MO, USA; C6628), E-64d (Sigma, E8640) and Pepstain A (Sigma, P4265) were dissolved in DMSO (Sigma, D2650). Acridine orange (Sigma, A6014) and 3-MA (Meilunbio, MB5063) were dissolved in PBS. MG132 (Meilunbio, MB5137), Etoposide (Meilunbio, MB1102-S) and CHX (Meilunbio, MB2208) were dissolved in DMSO. LC3 (MBL, Beijing, China; PM036) was bought for immunoblot or immunofluorescence analysis. Antibodies including AKT, phosphorylation of AKT (Ser473; 4060), MTOR, phosphorylation of MTOR (Ser2443; 5535), PARP (116 and 89 kd; 9542S), BAX (5023), phosphorylation of p53 (Ser15; 9286) and GAPDH (Santa Cruz Biotechnology(CA, USA) 5174) were purchased from CST (Beverly, MA, USA). Cleaved-PARP antibody was purchased from Sigma (SAB4500487). p53 (SC-126) and LAMP1 (SC-5570) were purchased from Santa Cruz Biotechnology (CA, USA) for immunoblot or immunofluorescence analyses.

### Cell proliferation assays

Overall, 1.0 **×** 10^4^ cells were seeded in 96-well plates and incubated at 37 °C for 24 h, followed by treatment with BA at indicated concentrations for another 48 h. After BA treatment, CCK8 reagents (Dojindo, Kamimashiki-gun, Kumamoto, Japan; CK04) were added and co-incubated for 1 h. The absorbance value was then measured at wavelength 450 nm.

### Detection of apoptosis

Overall, 1.0 × 10^6^ cells were seeded in six-well plates and incubated at 37°C for 24 –48 h, and then the fresh medium containing BA or the mixture of BA and autophagy inhibitors at indicated concentration for 48 h were replaced. According to the recommended protocol, the apoptosis rates were analyzed by Annexin-V-FITC and PI double-staining method based on the apoptosis detection kit (Beyotime Biotechnology, Shanghai, China) through flow cytometry (BD Biosciences, San Jose, CA, USA).

### DAPI staining assay

Cells were cultured in six-well plates with indicated concentrations of BA for 48 h. Then, cells were washed with PBS once and fixed by 4% paraformaldehyde for 15 min. Subsequently, DAPI diluted by PBS (1 : 20) was added into each well for 10 min following by washing with PBS for 10 min twice, and the blue-stained nuclei in cells were observed with fluorescence microscopy immediately.

### Transmission electron microscopy

For TEM observation, cells were treated with DMSO and 60 *μ*M BA for 48 h. The methods of TEM detection was previously described in detail.^78^

### Detection of AVOs

Cells were seeded on coverslips in 24-well plates and were allowed to attach by overnight incubation. Following treatment with DMSO (control) or BA at indicated concentrations, cells were stained with 1 *μ*g/ml acridine orange in PBS for 15 min, washed with PBS and examined under high-magnification fluorescence microscope (Olympus Optical Co., Hamburg, Germany).

### Transfection

All siRNAs were chemically synthesized by GenePharma company (Shanghai, China). The sequences of the siRNAs used are listed in Supplementary Table 1. The siRNAs were transfected into the indicated cells using Lipofectamine RNAiMAX reagent (Invitrogen, Carlsbad, CA, USA) according to the manufacturer's instructions. Cells were then collected and subjected to analysis 24–72 h after transfection. The experiments were repeated at least three times. Vectors were transfected by using Lipofectamine 2000 reagents (Invitrogen) according to the manufacturer's instructions for 48 h, and then cells were incubated with BA at indicated concentrations.

### GFP-LC3 and GFP-RFP-LC3 spot detection

First, cells were seeded on coverslips in 24-well plates and were allowed to attach by overnight incubation. GFP-LC3 or GFP-RFP-LC3 vectors were transfected in HCT116, SW480 and HT29 cells, respectively, for 24 h, following treatment with DMSO (control), or BA for 48 h. After depletion of the medium, cells were washed with PBS three times, fixed by 4% paraformaldehyde for 15 min, washed with PBS three times and photographed with a high-magnification fluorescence microscope (Olympus Optical Co.).

### Immunofluorescence and colocalization analysis

Cells were seeded on coverslips in 24-well plates and were allowed to attach by overnight incubation following treatment with DMSO (control) or BA at indicated concentrations for 48 h. Cells were fixed in 4% paraformaldehyde at 4 °C for 15 min, permeabilized with 0.01% Triton X-100 for 5 min and then blocked with 10% BSA for 1 h. Cells were incubated with primary Ab (rabbit anti-human LC3 or/and LAMP1, diluted 1 : 200, respectively) overnight, and then incubated with secondary Ab (Alexa fluor 594 goat anti-rabbit or/and Alexa fluor 488 goat anti-mouse) for 1 h. Unbound Ab was removed with PBS; thereafter, cell nuclei were stained with DAPI (1 : 20). The samples were examined under a high-magnification fluorescence microscope (Olympus Optical Co.).

### Quantitative RT-PCR

Total RNA was extracted from cells treated with BA at indicated concentration or was transfected with siRNA or vectors by using Trizol reagent (Invitrogen). cDNA reverse transcription and qRT-PCR were performed as the instruction indication of Reverse Transcription PrimeScript 1st Stand cDNA Synthesis kit (TaKaRa, Otsu, Japan) and quantitative PCR reagents SYBR PremixEx TaqTM (TaKaRa). The relative expression of genes was calculated with the 2^−ΔΔCt^ method. The sequences of the primers used are presented in Supplementary Table 2.

### MicroRNA RT-PCR

Total RNA was extracted from cultured three CRC cells by using Trizol reagent (Invitrogen). The expression level of matured miRNAs was analyzed by stem–loop reverse transcription followed by PrimeScript RT Reagent Kit (Takara). MicroRNA expression was normalized to the endogenous reference gene, U6. qPCR was performed by using SYBR PremixEx Taq-II (TaKaRa). The 2^−^^ΔΔCT^ method was used to quantify the expression changes of target genes. Three independent experiments were carried out. The primers used were listed in Supplementary Tables 3 and 4.

### Immunoblot analysis

After treatment with BA, the cells were collected, washed with ice-cold PBS and then extracted into ice-cold protein lysates RIPA or SDS buffer (Beyotime Biotechnology, Shanghai, China) with 1 mM PMSF (Hangzhou Beyotime Biotechnology). After being sonicated, the cells were centrifuged at 12 000 × *g* for 15 min at 4°C. The protein concentration was determined using a bicinchoninic acid protein assay kit (Bio-Rad, Hercules, CA, USA) according to the manufacturer’s instructions. Equivalent amounts of protein samples and 4 *μ*l pre-stained protein markers were uploaded, separated by 8–15% polyacrylamide gel electrophoresis, and then were transferred onto PVDF membranes (Millipore, Bedford, MA, USA). The membranes were blocked in 5% (w/v) skimmed milk, or in 5% (w/v) BSA only for p-AKT, p-MTOR and p-p53 protein, dissolving in TBS-T at room temperature for 2 h and then incubated with dilution of appropriate antibodies according to antibody specification overnight at 4 °C, followed by incubation with goat anti-rabbit or anti-mouse horseradish peroxidase-conjugated secondary antibody at room temperature for 1 h. The blots were visualized by using chemiluminescent substrates (Millipore).

### Statistical analysis

Multiple comparisons of IC50 were performed by repeated measures ANOVA and Bonferroni's test using GraphPad Prism 5 Software (La Jolla, CA, USA). Student’s (two-tailed) *t*-test was also conducted. *P-*value<0.05 was marked as statistically significant. *P*-value<0.01 was indicated as highly statistically significant. *P-*value<0.001 was indicated as extremely statistically significant difference. Values are the means of three independent experiments (means±SD).

## Publisher’s Note

Springer Nature remains neutral with regard to jurisdictional claims in published maps and institutional affiliations.
