# Supplementary material for: Overaccumulation of p53-mediated autophagy protects against betulinic acid-induced apoptotic cell death in colorectal cancer cells
Source: Cell Death Dis. 2017 Oct 5;8(10):e3087–. doi: 10.1038/cddis.2017.485 (PMC5682653; doi:10.1038/cddis.2017.485)
Supplement: Supplementary Figures [file cddis2017485x1.pdf]

Figure S1

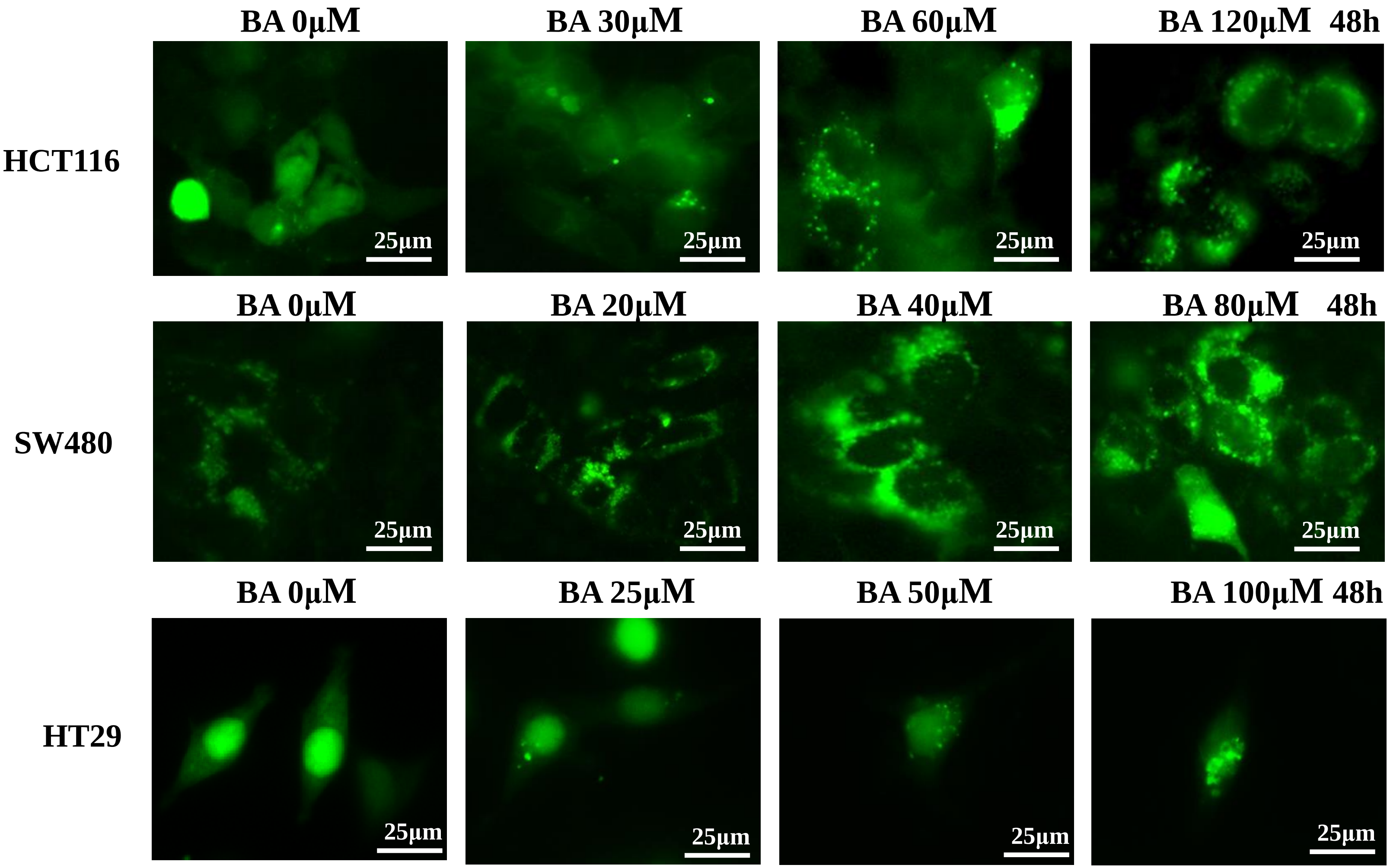

BA 0μM
BA 30μM
BA 60μM
BA 120μM 48h
HCT116
BA 0μM
BA 20μM
BA 40μM
BA 80μM 48h
SW480
BA 0μM
BA 25μM
BA 50μM
BA 100μM 48h
HT29
25μm

Figure S2

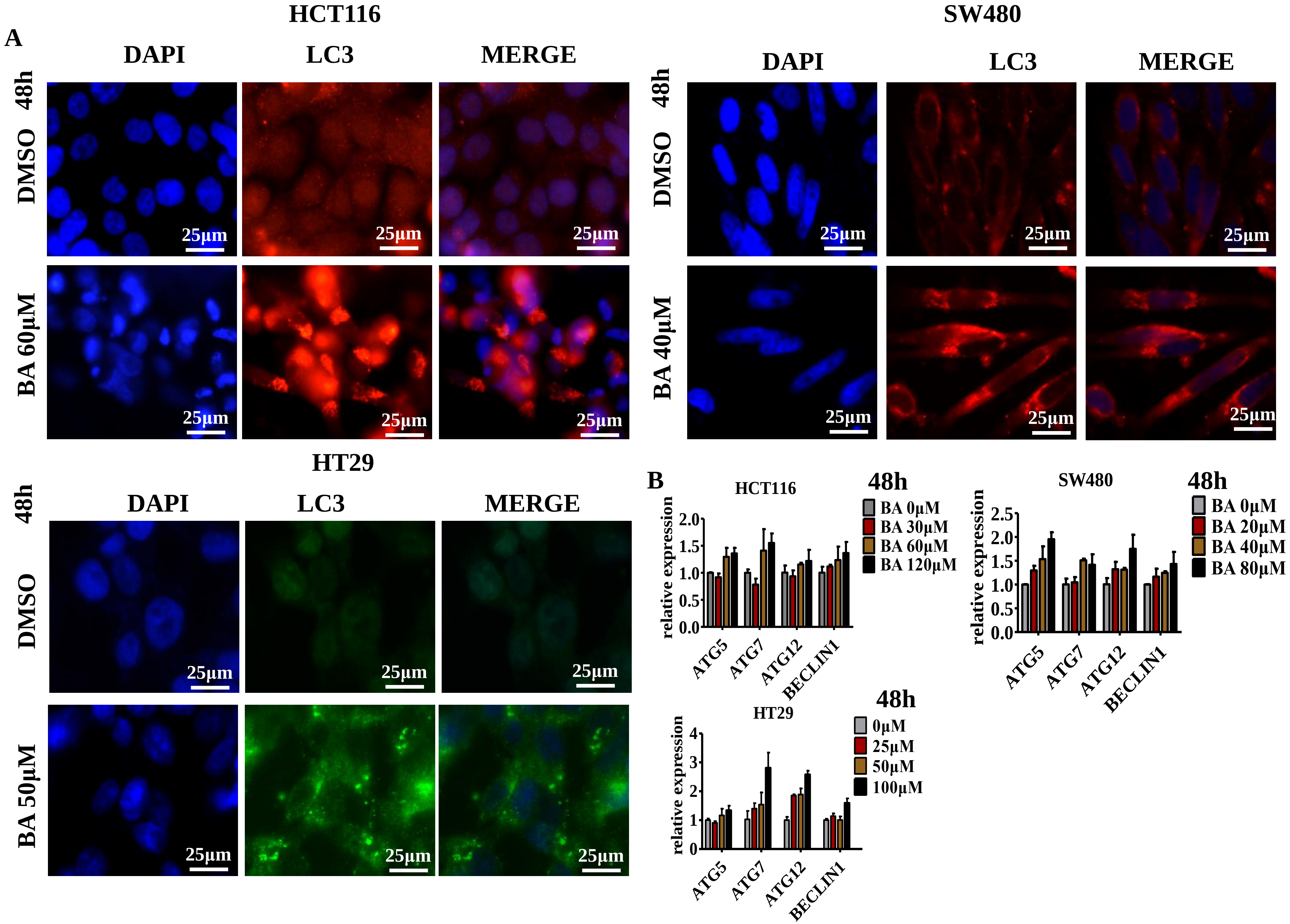
A
HCT116
DAPI
LC3
MERGE
DMSO 48h
BA 60μM
25μm
SW480
DAPI
LC3
MERGE
DMSO 48h
BA 40μM
25μm
HT29
DAPI
LC3
MERGE
DMSO 48h
BA 50μM
25μm
B
HCT116
48h
BA 0μM
BA 30μM
BA 60μM
BA 120μM
relative expression
ATG5
ATG7
ATG12
BECLIN1
SW480
48h
BA 0μM
BA 20μM
BA 40μM
BA 80μM
relative expression
ATG5
ATG7
ATG12
BECLIN1
HT29
48h
0μM
25μM
50μM
100μM
relative expression
ATG5
ATG7
ATG12
BECLIN1

Figure S3

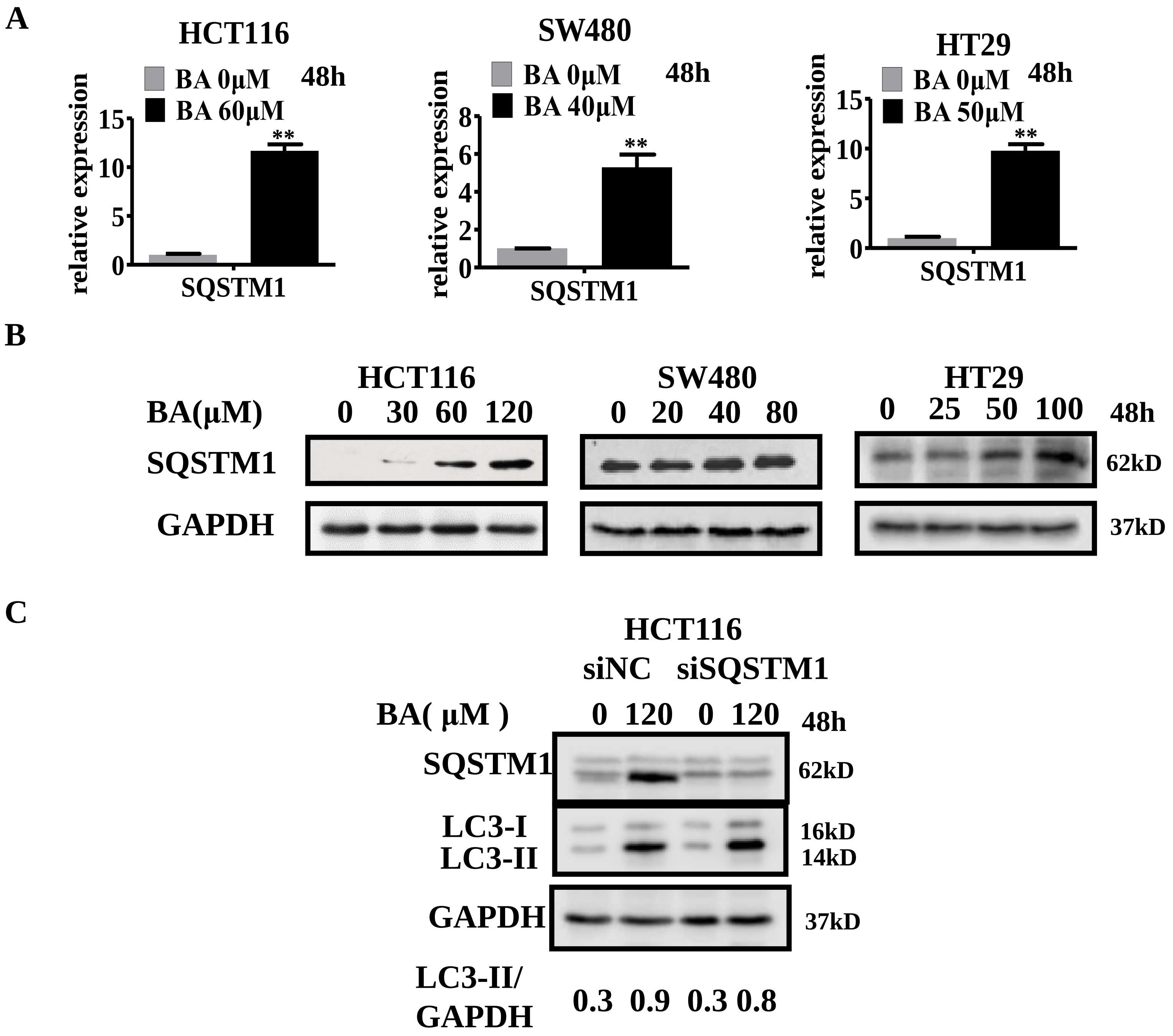

A
HCT116
BA 0μM
BA 60μM
48h
relative expression
15
10
5
0
**
SQSTM1
SW480
BA 0μM
BA 40μM
48h
relative expression
8
6
4
2
0
**
SQSTM1
HT29
BA 0μM
BA 50μM
48h
relative expression
15
10
5
0
**
SQSTM1
B
HCT116
SW480
HT29
BA(μM)
0 30 60 120
0 20 40 80
0 25 50 100
48h
SQSTM1
GAPDH
62kD
37kD
C
HCT116
siNC siSQSTM1
BA(μM)
0 120 0 120
48h
SQSTM1
LC3-I
LC3-II
GAPDH
62kD
16kD
14kD
37kD
LC3-II/
GAPDH
0.3 0.9 0.3 0.8

Figure S4

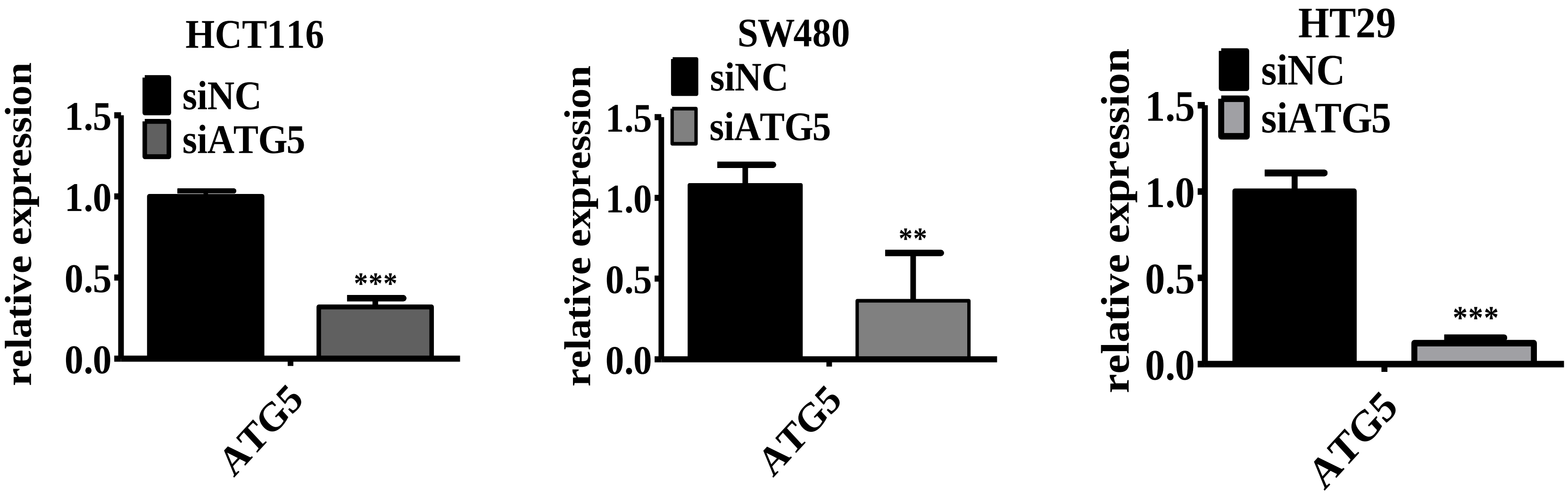

HCT116
siNC
siATG5
relative expression
1.5
1.0
0.5
0.0
***
ATG5
SW480
siNC
siATG5
relative expression
1.5
1.0
0.5
0.0
**
ATG5
HT29
siNC
siATG5
relative expression
1.5
1.0
0.5
0.0
***
ATG5

Figure S5

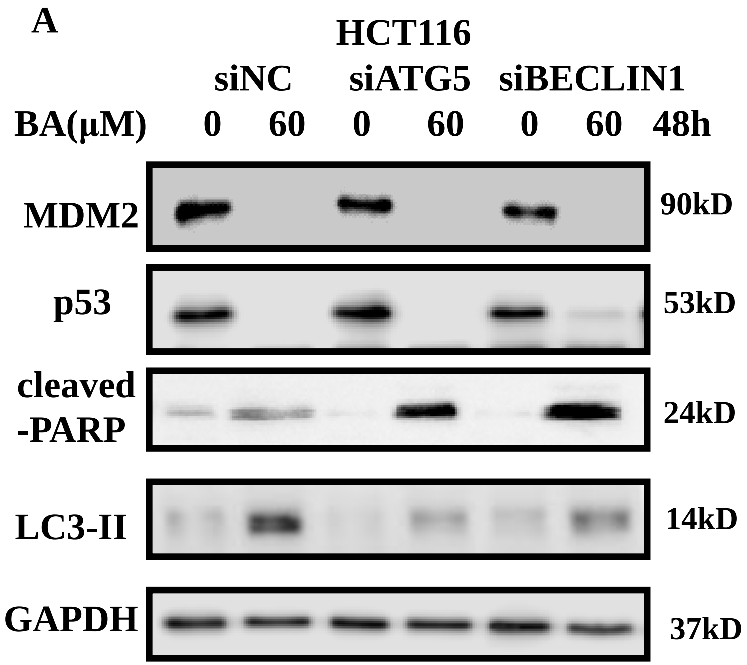

A
HCT116
siNC
siATG5
siBECLIN1
BA(μM)
0
60
0
60
0
60
48h
MDM2
p53
cleaved
-PARP
LC3-II
GAPDH
90kD
53kD
24kD
14kD
37kD

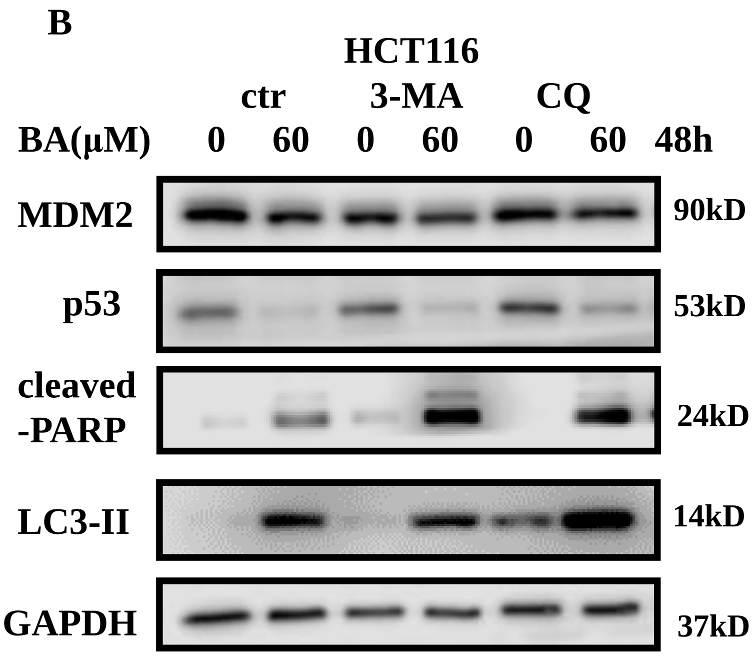

B
HCT116
ctr
3-MA
CQ
BA(μM)
0
60
0
60
0
60
48h
MDM2
p53
cleaved
-PARP
LC3-II
GAPDH
90kD
53kD
24kD
14kD
37kD

Figure S6

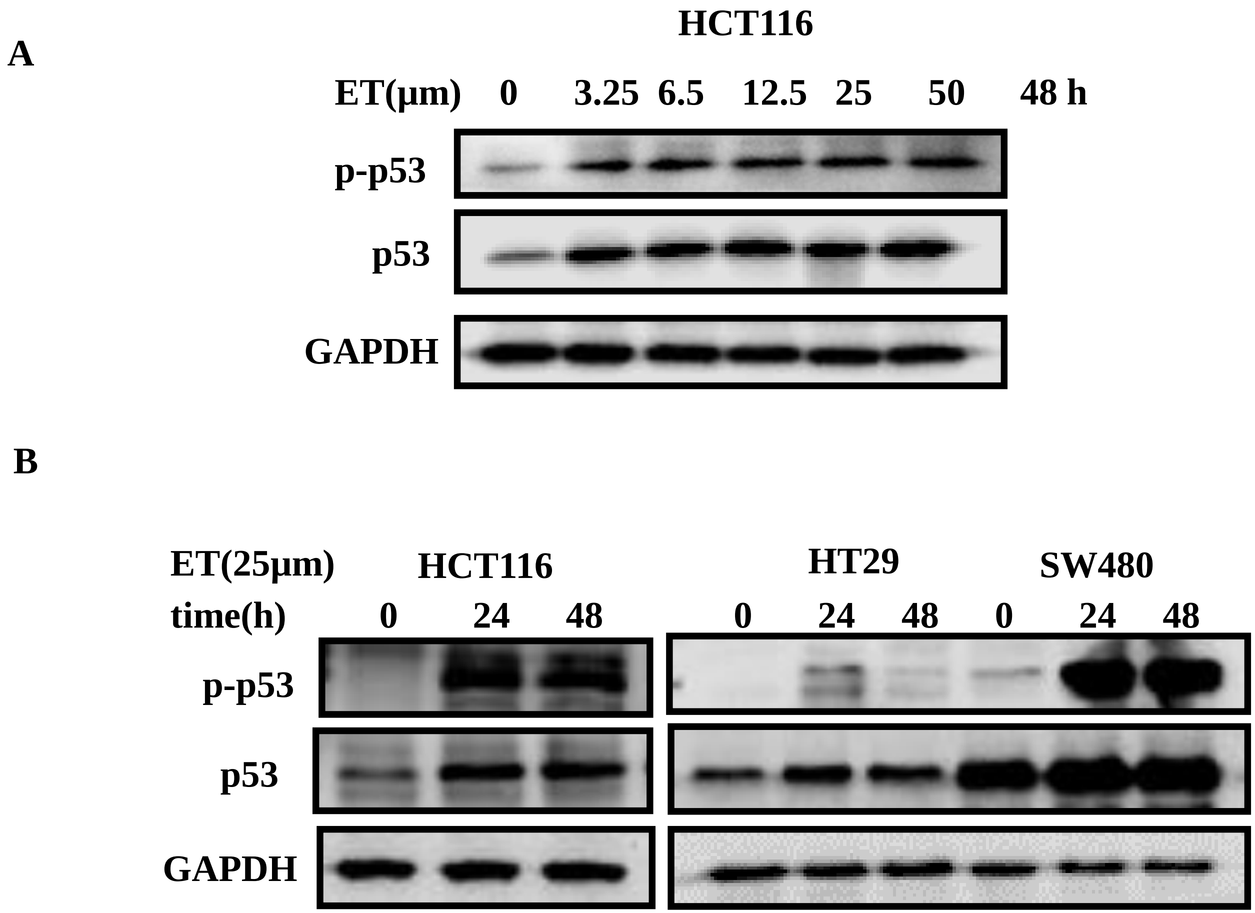
A
HCT116
ET(μm) 0 3.25 6.5 12.5 25 50 48 h
p-p53
p53
GAPDH
B
ET(25μm)
time(h)
HCT116
0 24 48
HT29
0 24 48
SW480
0 24 48
p-p53
p53
GAPDH

Figure S7

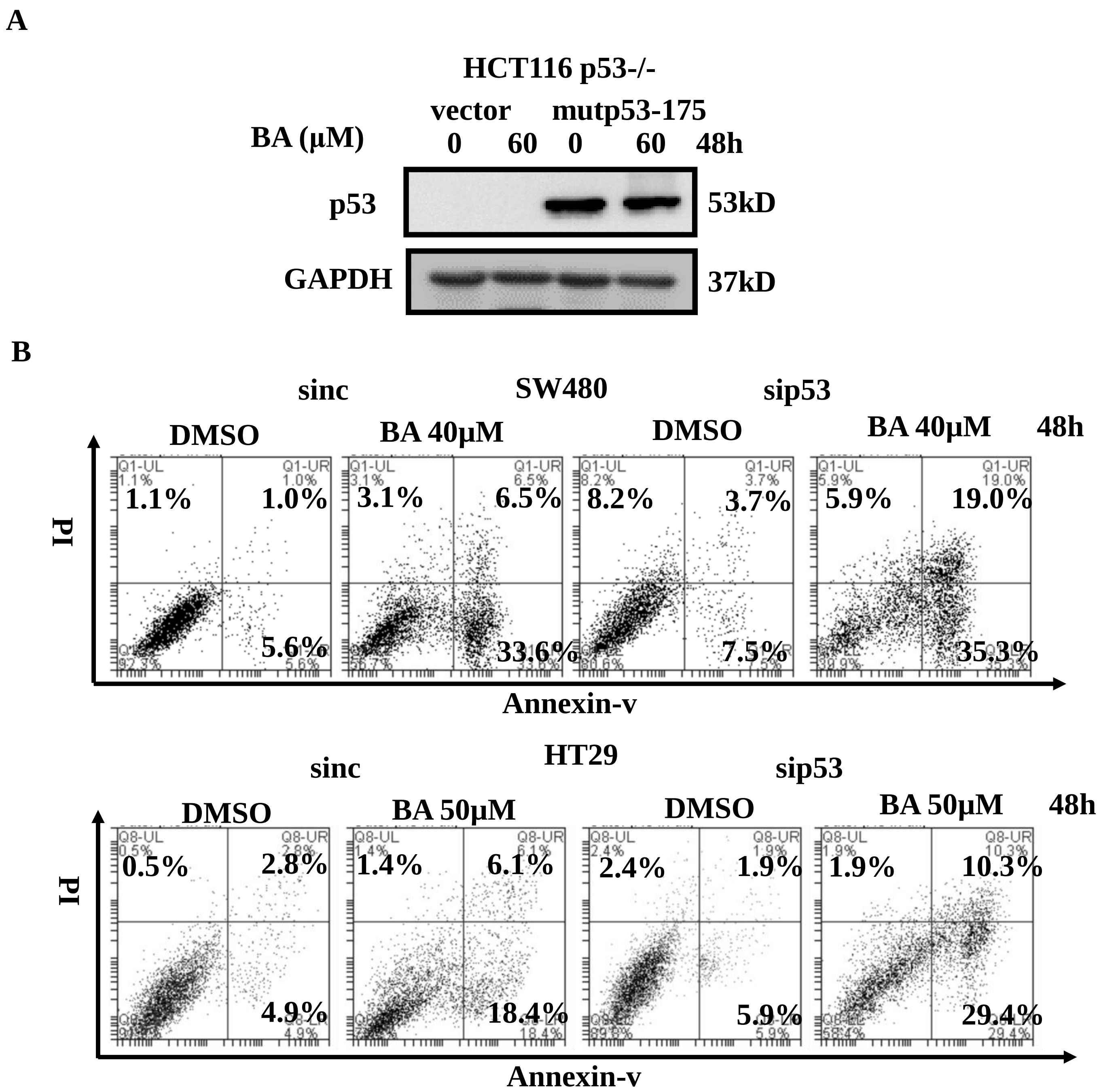
A
HCT116 p53-/-
vector
mutp53-175
BA (μM)
0
60
0
60
48h
p53
53kD
GAPDH
37kD
B
sinc
SW480
sip53
DMSO
BA 40μM
DMSO
BA 40μM
48h
PI
1.1%
1.0%
5.6%
3.1%
6.5%
33.6%
8.2%
3.7%
7.5%
5.9%
19.0%
35.3%
Annexin-v
sinc
HT29
sip53
DMSO
BA 50μM
DMSO
BA 50μM
48h
PI
0.5%
2.8%
4.9%
1.4%
6.1%
18.4%
2.4%
1.9%
5.9%
1.9%
10.3%
29.4%
Annexin-v

Figure S8

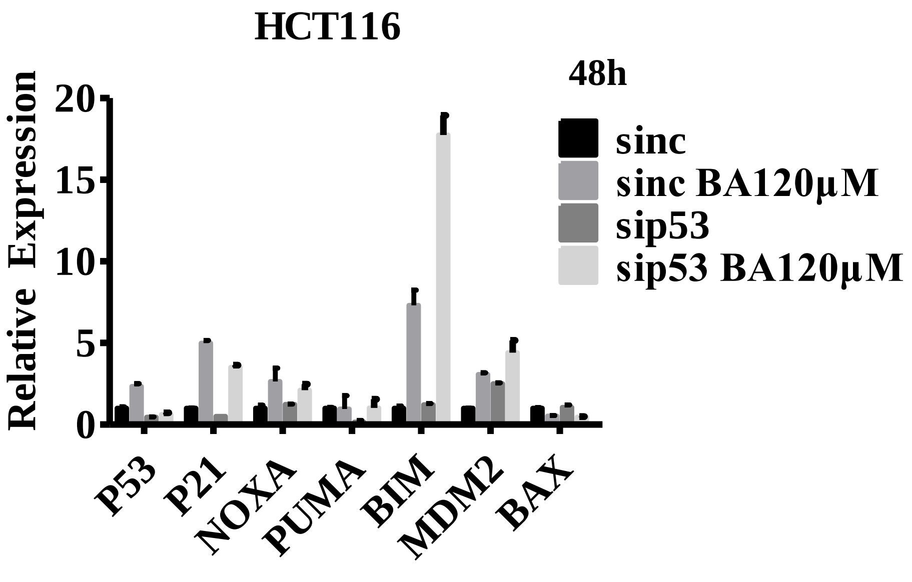
HCT116
48h
Relative Expression
20
15
10
5
0
sinc
sinc BA120μM
sip53
sip53 BA120μM
P53
P21
NOXA
PUMA
BIM
MDM2
BAX

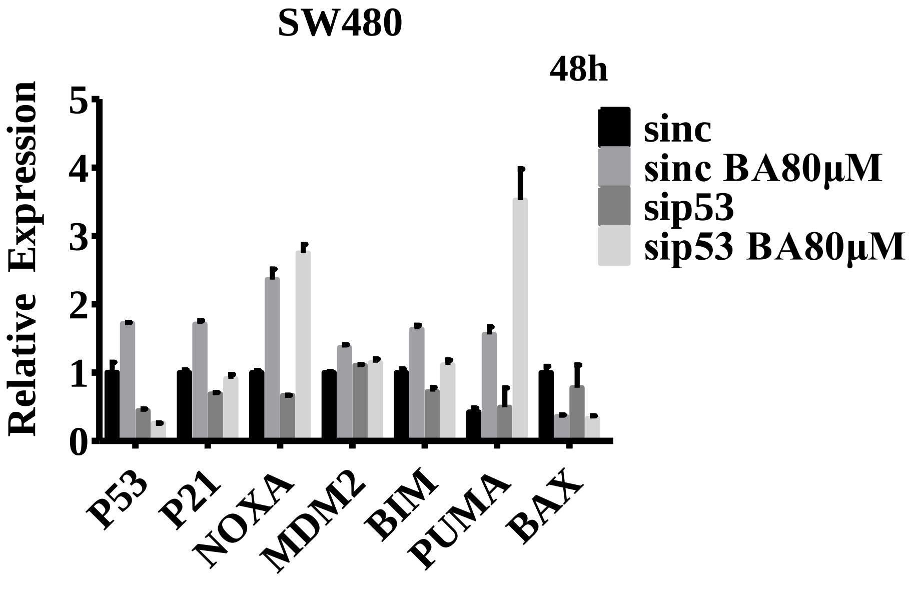
SW480
48h
Relative Expression
5
4
3
2
1
0
sinc
sinc BA80μM
sip53
sip53 BA80μM
P53
P21
NOXA
MDM2
BIM
PUMA
BAX

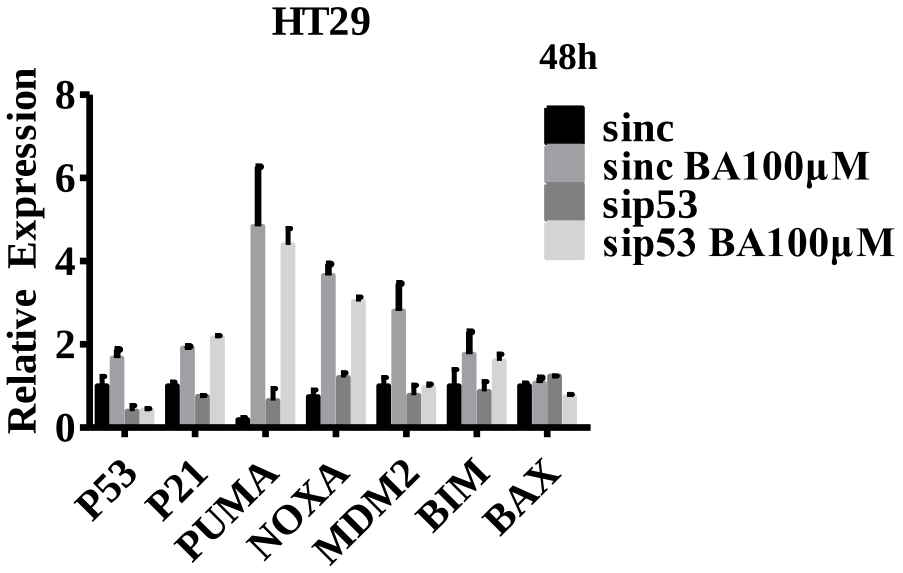
HT29
48h
Relative Expression
8
6
4
2
0
sinc
sinc BA100μM
sip53
sip53 BA100μM
P53
P21
PUMA
NOXA
MDM2
BIM
BAX

Figure S9

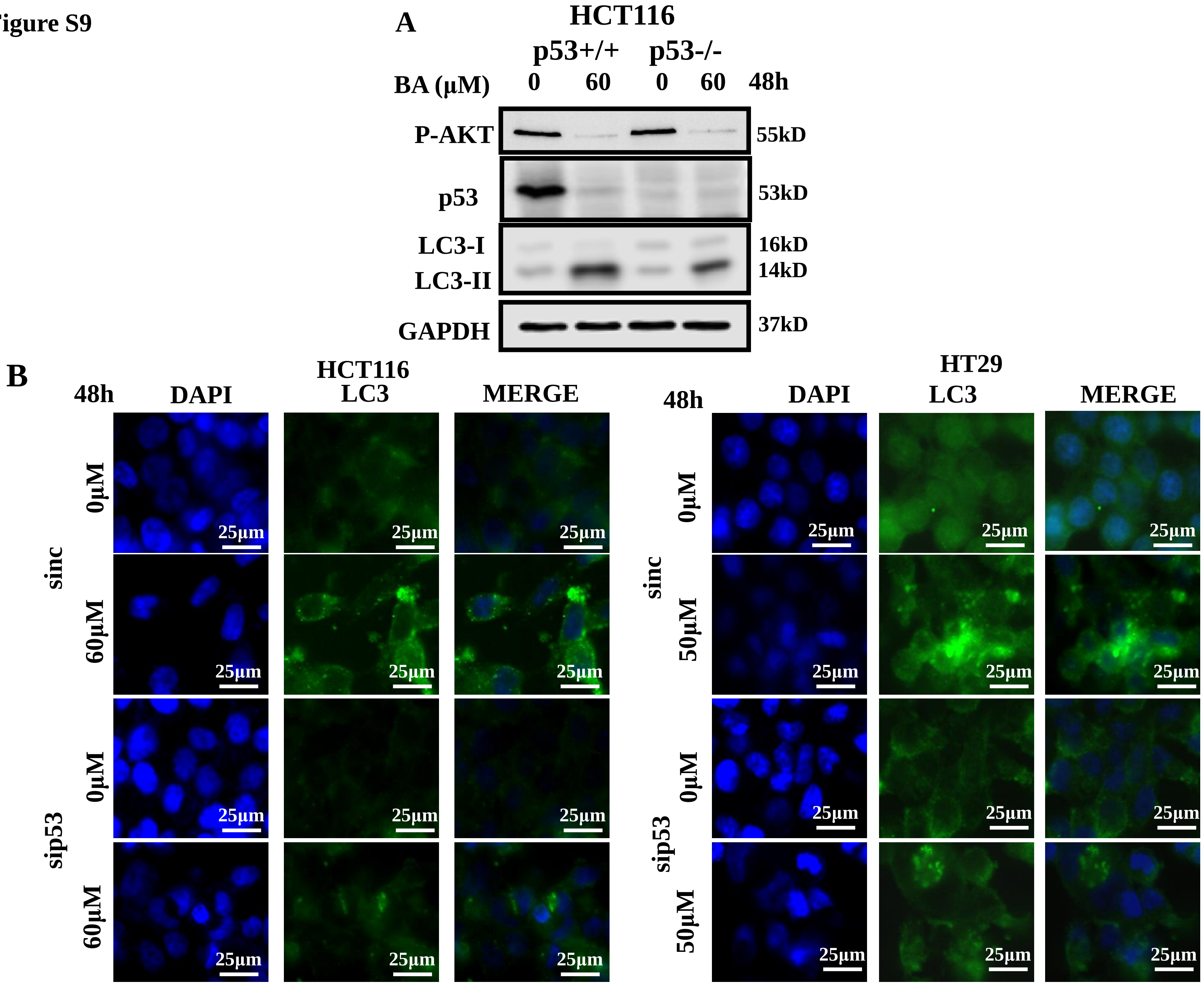
A
HCT116
p53+/+
p53-/-
BA (μM)
0
60
0
60
48h
P-AKT
55kD
p53
53kD
LC3-I
LC3-II
16kD
14kD
GAPDH
37kD
B
HCT116
48h
DAPI
LC3
MERGE
sinc
sip53
0μM
60μM
25μm
HT29
48h
DAPI
LC3
MERGE
sinc
sip53
0μM
50μM
25μm

Figure S10

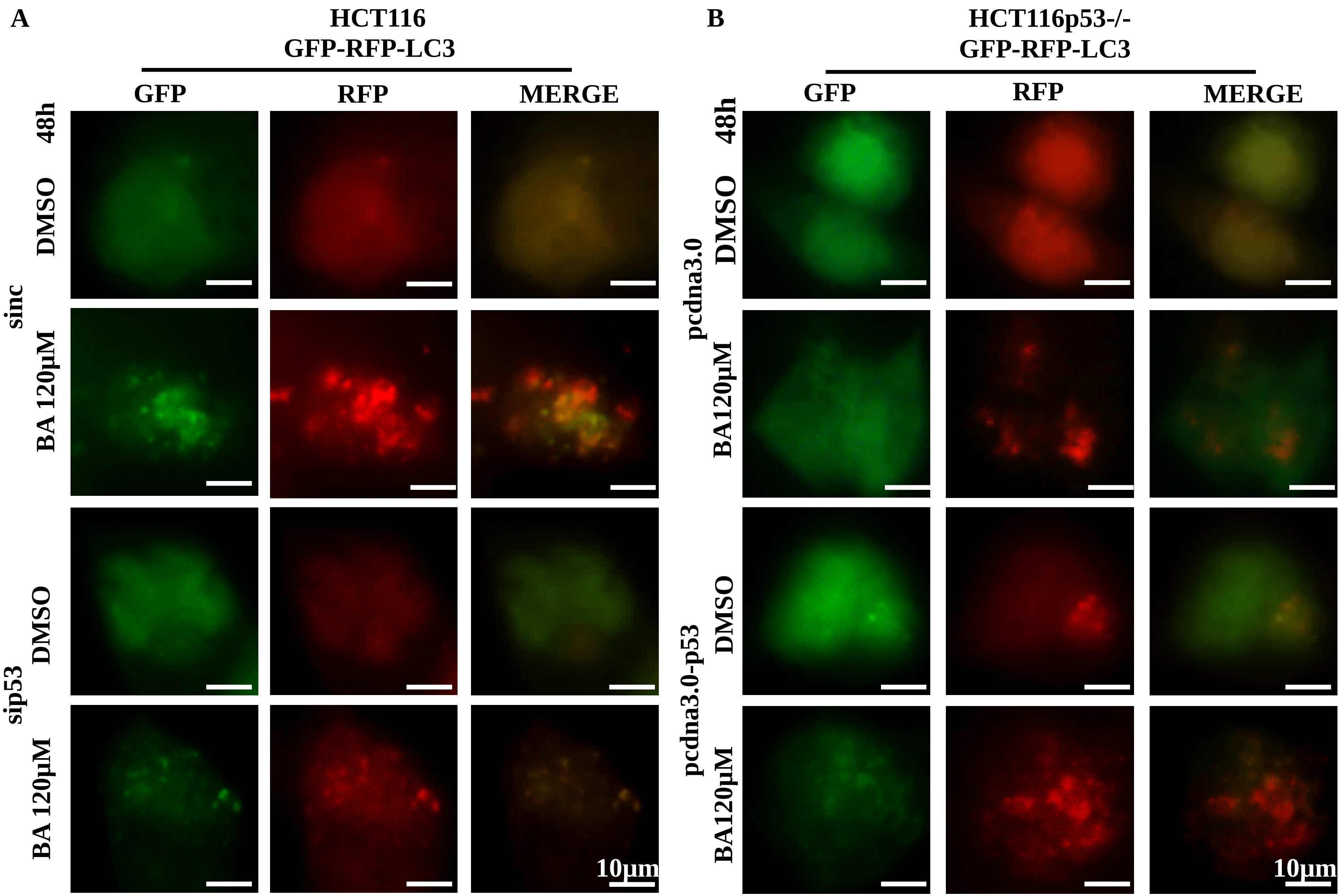

A
HCT116
GFP-RFP-LC3
GFP
RFP
MERGE
48h
DMSO
sinc
BA 120μM
DMSO
sip53
BA 120μM
10μm
B
HCT116p53-/-
GFP-RFP-LC3
GFP
RFP
MERGE
48h
DMSO
pcdna3.0
BA120μM
DMSO
pcdna3.0-p53
BA120μM
10μm

Figure S11

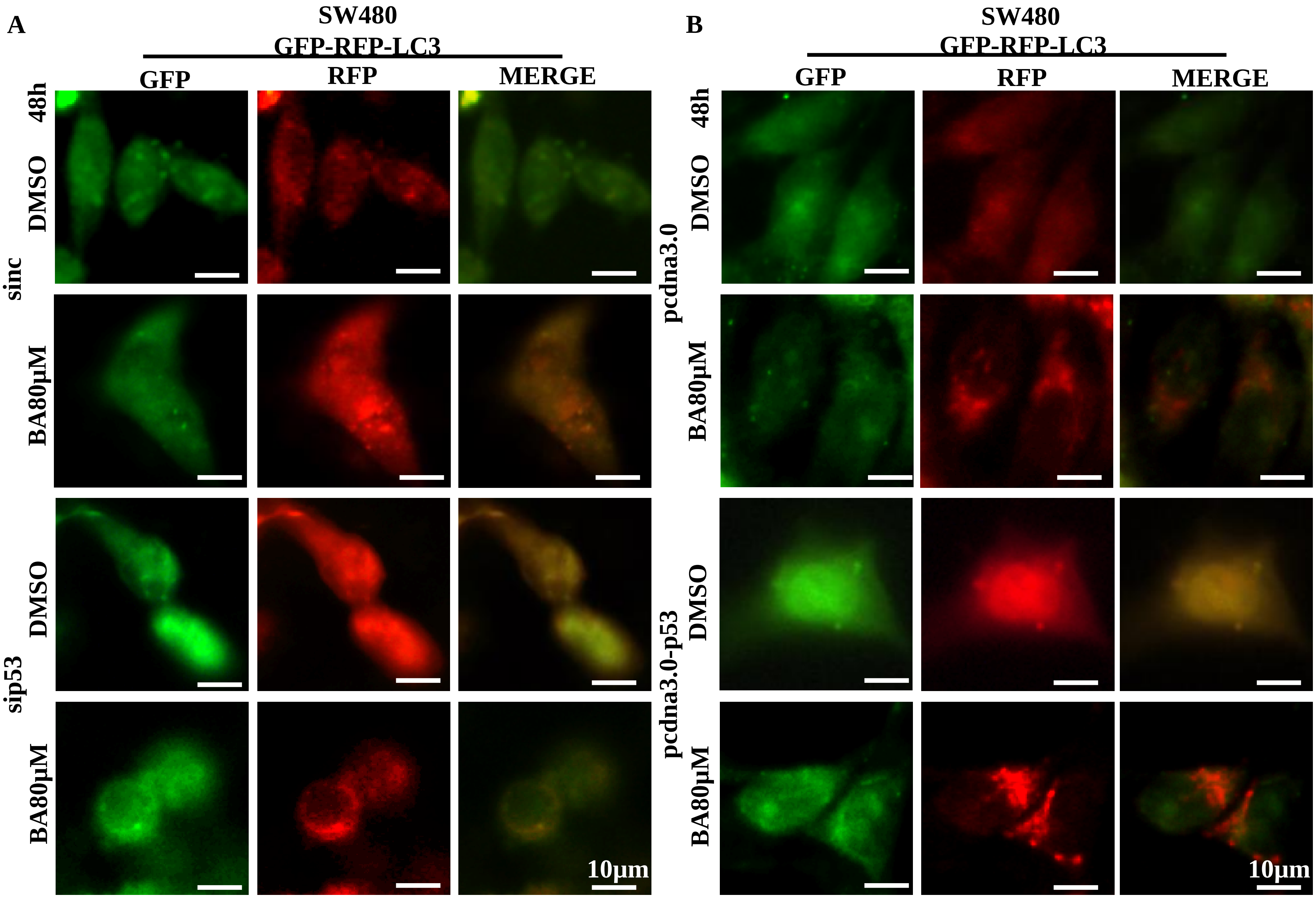
A
SW480
GFP-RFP-LC3
GFP
RFP
MERGE
48h
DMSO
BA80μM
sinc
sip53
10μm
B
SW480
GFP-RFP-LC3
GFP
RFP
MERGE
48h
DMSO
BA80μM
pcdna3.0
pcdna3.0-p53
10μm

Figure S12

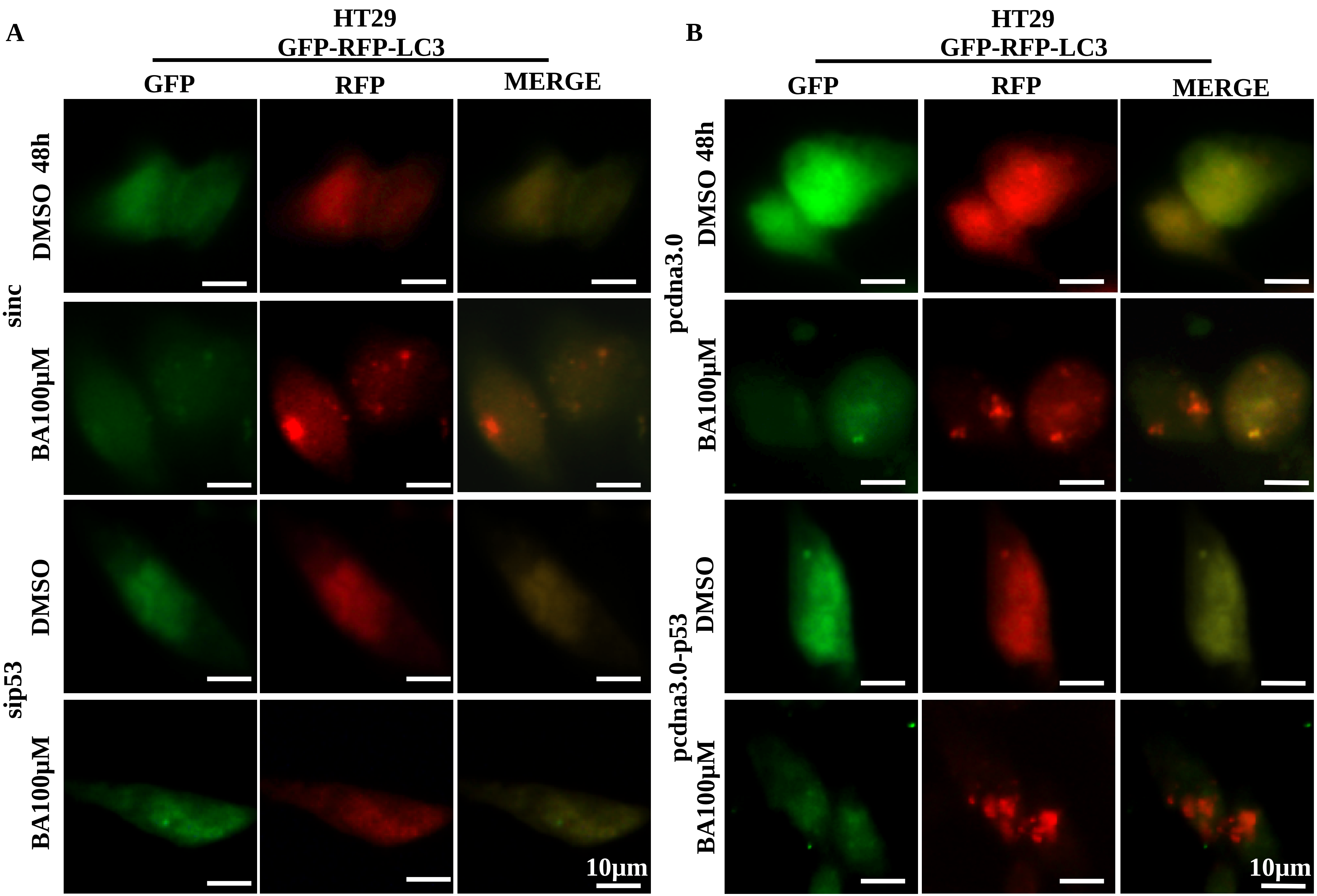
A
HT29
GFP-RFP-LC3
GFP
RFP
MERGE
sinc
DMSO 48h
BA100μM
sip53
DMSO
BA100μM
10μm
B
HT29
GFP-RFP-LC3
GFP
RFP
MERGE
pcdna3.0
DMSO 48h
BA100μM
pcdna3.0-p53
DMSO
BA100μM
10μm

Figure S13

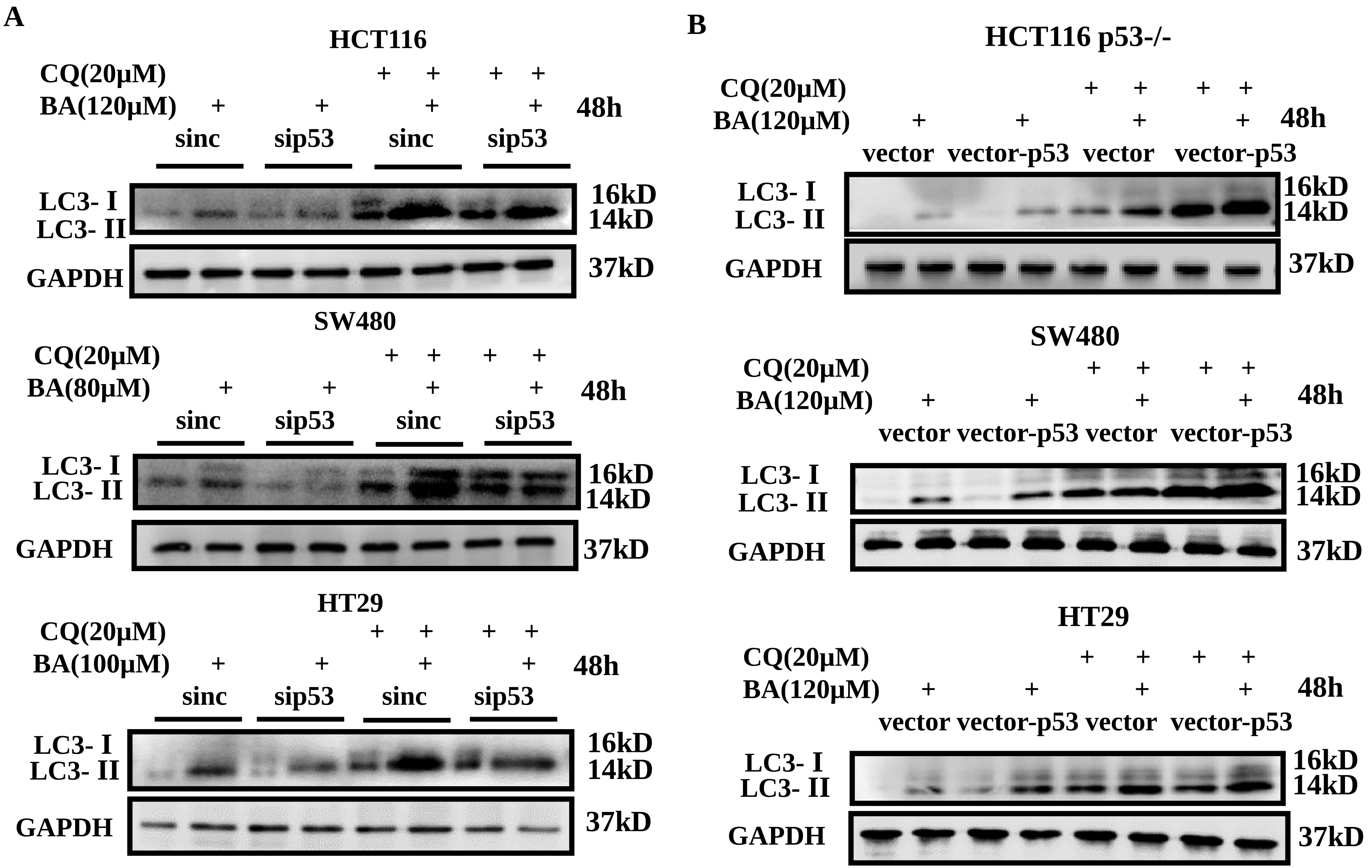
A
HCT116
CQ(20μM)
BA(120μM)
48h
sinc
sip53
sinc
sip53
LC3- I
LC3- II
GAPDH
16kD
14kD
37kD
SW480
CQ(20μM)
BA(80μM)
48h
sinc
sip53
sinc
sip53
LC3- I
LC3- II
GAPDH
16kD
14kD
37kD
HT29
CQ(20μM)
BA(100μM)
48h
sinc
sip53
sinc
sip53
LC3- I
LC3- II
GAPDH
16kD
14kD
37kD
B
HCT116 p53-/-
CQ(20μM)
BA(120μM)
48h
vector
vector-p53
vector
vector-p53
LC3- I
LC3- II
GAPDH
16kD
14kD
37kD
SW480
CQ(20μM)
BA(120μM)
48h
vector
vector-p53
vector
vector-p53
LC3- I
LC3- II
GAPDH
16kD
14kD
37kD
HT29
CQ(20μM)
BA(120μM)
48h
vector
vector-p53
vector
vector-p53
LC3- I
LC3- II
GAPDH
16kD
14kD
37kD

Figure S14

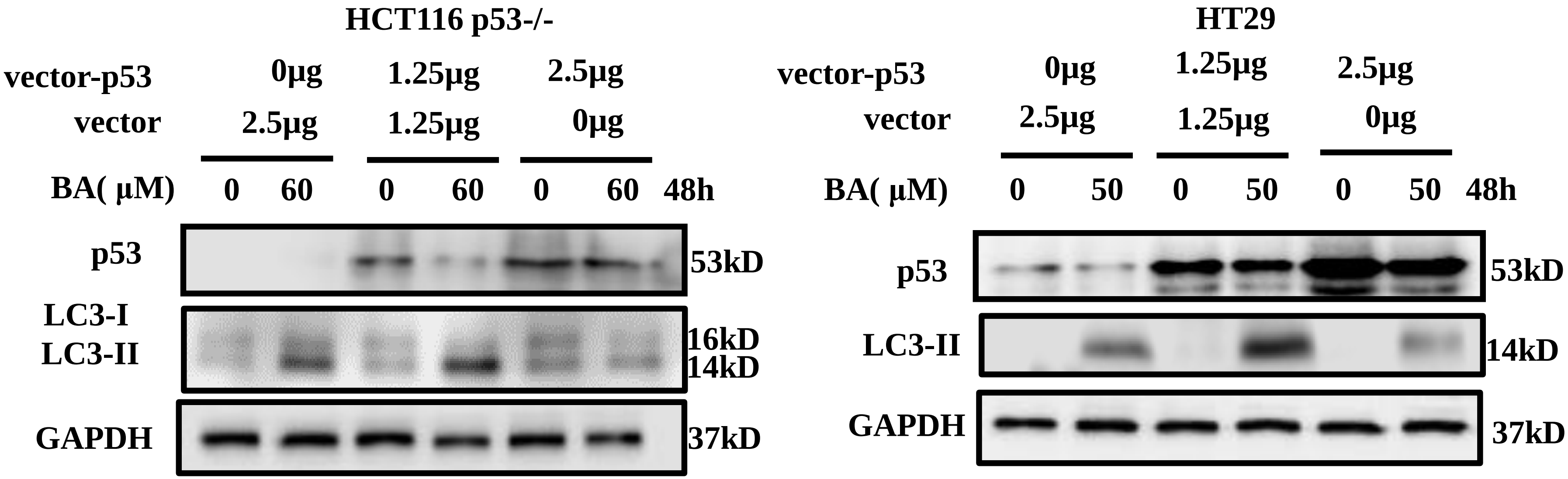

HCT116 p53-/-
vector-p53 0μg 1.25μg 2.5μg
vector 2.5μg 1.25μg 0μg
BA(μM) 0 60 0 60 0 60 48h
p53
53kD
LC3-I
LC3-II
16kD
14kD
GAPDH
37kD
HT29
vector-p53 0μg 1.25μg 2.5μg
vector 2.5μg 1.25μg 0μg
BA(μM) 0 50 0 50 0 50 48h
p53
53kD
LC3-II
14kD
GAPDH
37kD

Figure S15

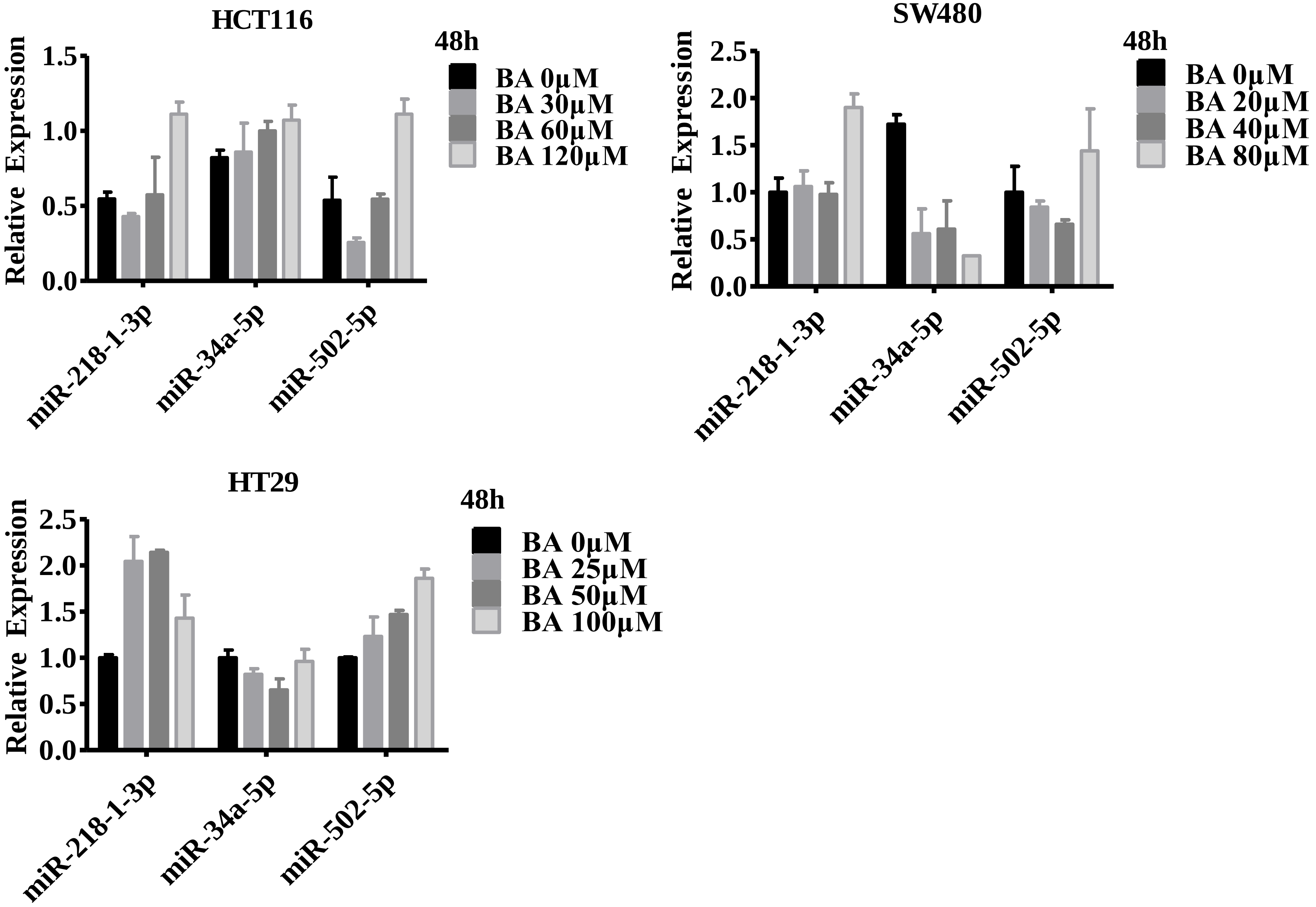
HCT116
48h
BA 0μM
BA 30μM
BA 60μM
BA 120μM
Relative Expression
1.5
1.0
0.5
0.0
miR-218-1-3p
miR-34a-5p
miR-502-5p
SW480
48h
BA 0μM
BA 20μM
BA 40μM
BA 80μM
Relative Expression
2.5
2.0
1.5
1.0
0.5
0.0
miR-218-1-3p
miR-34a-5p
miR-502-5p
HT29
48h
BA 0μM
BA 25μM
BA 50μM
BA 100μM
Relative Expression
2.5
2.0
1.5
1.0
0.5
0.0
miR-218-1-3p
miR-34a-5p
miR-502-5p
