# Supplementary material for: Overaccumulation of p53-mediated autophagy protects against betulinic acid-induced apoptotic cell death in colorectal cancer cells
Source: Cell Death Dis. 2017 Oct 5;8(10):e3087–. doi: 10.1038/cddis.2017.485 (PMC5682653; doi:10.1038/cddis.2017.485)
Supplement: Supplementary Figure Legends [file cddis2017485x2.docx]

**Supplementary Figure legends**

**Figure S1.** HCT116, SW480 and HT29 cells were seeded on the slides in 24-well plates, transfected with GFP-LC3 for 48h next day, and then treated with BA at the indicated concentrations for another 48h. GFP-LC3 puncta was observed under high magnification fluorescence microscopy.

**Figure S2.** **A**. HCT116, SW480 and HT29 cells were treated with BA (60μM for HCT116, 40μM for SW480, 50μM for HT29) for 48h and then the accumulation of endogenous LC3 was analyzed by indirect immunofluorescence method. Nuclei was stained with DAPI. DMSO was the control group. **B**. HCT116, SW480 and HT29 cells were treated with the indicated concentrations of BA for 48h. Autophagy genes (ATG5, ATG7, ATG12 and BECLIN1) were determined by qRT-PCR. DMSO was the control group. GAPDH was internal control.

**Figure S3. A**. HCT116, SW480 and HT29 cells were treated with BA at the indicated concentrations for 48h. qRT-PCR were performed by using SQSTM1 specific primer to determine SQSTM1 mRNA levels. GAPDH was internal control. DMSO was the control group. **B**. HCT116, SW480 and HT29 cells were treated with the indicated concentrations of BA for 48h and total protein were extracted and blotted with SQSTM1 and GAPDH antibodies. GAPDH was internal control. DMSO was the control group. **C**. HCT116 cells were transfected with siRNA specific to negative control and SQSTM1 for 24h, and then incubated with the indicated concentrations of BA for another 48h. DMSO was the control group. Total protein was extracted and blotted with the indicated protein antibodies. GAPDH was internal control. The ratio between GAPDH and LC3-II was calculated by Image J.

**Figure S4.** Small interference ATG5 and negative control were transfected in HCT116, SW480 and HT29 cells for 24h. The mRNA levels of ATG5 were determined by qRT-PCR. GAPDH was internal control. Data are shown as means ± STD. ** represents p<0.01. *** p<0.001. DMSO was the control group.

**Figure S5. A**. HCT116 cells were transfected with siRNA targeting ATG5 or BECLIN 1 for 24h and then incubated with the indicated concentrations of BA for another 48h. MDM2, p53, cleaved-PARP, LC3 and internal control (GAPDH) were detected by immunoblot. DMSO was the control group. **B**. HCT116 cells were treated with 5mM 3-MA or 20μM CQ in company with BA or control for 48h, and then MDM2, p53, LC3, GAPDH proteins were detected by immunoblot. DMSO was the control group.

**Figure S6. A**. HCT116 cells were treated with etoposide at the indicated concentrations for 48h, and then p53 and phosphorylation p53 at S15 was determined by immunoblot. GAPDH was internal control. DMSO was the control group. **B**. HCT116, HT29 and SW480 cells were treated with etoposide at 25µM for 0h, 24h and 48h respectively, and then p53 and phosphorylation p53 at S15 were determined by immunoblot. GAPDH was internal control. DMSO was the control group.

**Figure S7. A.** HCT116 p53-/- cells were transfected with mutp53-175 and control vector for 48h and then incubated with the indicated concentrations of BA for another 48h. p53 protein expression was determined by immunoblot. GAPDH was internal control. DMSO was the control group. **B.** p53 was knocked down by siRNA specific to p53 in SW480 and HT29 cells, and the cells were treated with BA at 40µM or 50µM for 48h. After that, cell apoptosis was determined by AV/PI double staining flow cytometry. DMSO was the control group.

**Figure S8.** HCT116, SW480 and HT29 cells were treated with BA at indicated concentrations in the present or absent of p53, and then the expression of p53 and p53 target genes were determined by qRT-PCR. DMSO was the control group. GAPDH was internal control.

**Figure S9. A**. HCT116 p53+/+ and p53-/- cells were treated with BA at the indicated concentrations for 48h, and then P-AKT, p53, LC3 and GAPDH proteins were detected by immunoblot. DMSO was the control group. GAPDH was internal control. **B**. HCT116 and HT29 cells were seeded on slides in 24-well plates, transfected with siRNA targeting to control or p53 gene for 24h and incubated with the indicated concentration of BA for another 48h. DMSO was the control group. LC3 puncta was detected by indirect immunofluorescence. Nucleus was stained with DAPI.

**Figure S10-S12. Left,** p53 was knocked down by siRNA specific to p53 for 24h in HCT116, SW480 and HT29 cells, and then the cells were treated with BA at the indicated concentrations for 48h after transfection with GFP-RFP-LC3 for 24h. GFP-LC3 puncta and RFP-LC3 puncta was observed by high magnification microscope fluorescence microscopy. DMSO was the control group. **Right,** HCT116 p53-/-, SW480 and HT29 cells were transfected with GFP-RFP-LC3 for 24h before transfection with p53-vector (p53-pcdna3.0) and control vector (pcdna3.0) for 24h. Then the cells were treated with BA at the indicated concentrations for 48h. GFP-LC3 puncta and RFP-LC3 puncta were observed by high magnification microscope fluorescence microscopy. DMSO was the control group.

**Figure S13. A.** p53 was knocked down by siRNA specific to p53 for 24h in HCT116, SW480 and HT29 cells, and then the cells were treated with BA at the indicated concentrations in company with or without CQ(20µM) for 48h respectively. LC3 and GAPDH were determined by immunoblot. **B.** HCT116 p53-/-, SW480 and HT29 cells were transfected with p53-vector (p53-pcdna3.0) and control vector (pcdna3.0) for 48h, and treated with BA in company with or without CQ(20µM) for 48h. After that, LC3 and GAPDH were determined by immunoblot. GAPDH was internal control. DMSO was the control group.

**Figure S14.** HCT116 p53-/- cells and HT29 cells were transfected with p53-vector or control vector at the indicated doses for 48h and then incubated with the indicated concentrations of BA for another 48h respectively. p53, LC3 protein and internal control (GAPDH) were determined with corresponding antibodies by immunoblot. DMSO was the control group.

**Figure S15.** HCT116, SW480 and HT29 cells were treated with the indicated concentrations of BA for 48h, and then total RNA was isolated. The expression levels of matured miRNAs were analyzed by stem-loop reverse transcription followed by quantitative PCR (qPCR). U6 was used as internal control. DMSO was the control group.

**Supplementary Tables**

**Supplementary Table 1. List of Small Interference RNA Sequences**

| **Gene name siRNA sense sequences** |
| --- |
| BECLIN 1 5'-AAGAUAGUGGCAGAAAAUCTT-'3  ATG5 5'-GACGUUGGUAACUGACAAATT-'3  SQSTM1 5'-GCAUUGAAGUUGAUAUCGATT-'3  P53 5'-GACUCCAGUGGUAAUCUACdTT-'3  NC 5'-UUCUCCGAACGUGUCACGUUU-'3 |

**Supplementary Table 2. List of qRT-PCR Primer Sequences**

| **Gene name Primer sequences** |
| --- |
| ATG5 F329:5'-AAGACCTTCTGCACTGTCCA-3'  R512: 5'-GAGTTTCCGATTGATGGCCC-3'  ATG7 F1839: 5'-TATGCCTCCAACCTCTCTTG-3'  R2083: 5'-AGATCTCAGCAGCTTGGGTT-3'  ATG12 F1667: 5'-TAGCAAGGCAAGATCCCCAA-3'  R1821: 5'-GGGTCTCACTCTGTTGCTCA-3'  BECLIN1 F331: 5'-AACCTCAGCCGAAGACTGAA-3'  R571: 5'-CCTCTAGTGCCAGCTCCTTT-3'  BAX F112:5'-GAGCTGCAGAGGATGATTGC-3'  R313:5'-CCAATGTCCAGCCCATGATG-3'  NOXA F506:5'-GCCCTTGGAAACGGAAGATG-3'  F716:5'-CAGCGGTAATCTTCGGCAAA-3'  P21 F990: 5'-ATGAAATTCACCCCCTTTCC-3'  R1163: 5'-CCCTAGGCTGTGCTCACTT-3'  P53 F1359: 5'-GTTCCGAGAGCTGAATGAGG-3'  R1497: 5'-TCTGAGTCAGGCCCTTCTGT-3'  SQSTM1 F842: 5'-CCAGAGAGTTCCAGCACAGA-3'  R1010: 5'-CCGACTCCATCTGTTCCTCA-3'  MDM2 F659: 5'-AGCAGGAATCATCGGACTCA-3'  R877: 5'-TGTGGCGTTTTCTTTGTCGT-3'  BIM F102: 5'-CTCCCTACAGACAGAGCCAC-3'  R261: 5'-GTCTTCGGCTGCTTGGTAAT-3'  GAPDH F833: 5'-ACCTGACCTGCCGTCTAGAA-3'  R1060: 5'-TCCACCACCCTGTTGCTGTA-3' |

**Supplementary Table 3. List of MicroRNA reverse transcription primer sequences**

Hsa-miR-502-5p 5'- GTCGTATCCAGTGCGTGTCGTGGAGTCGG

CAATTGCACTGGATACGAC TAGCAC -'3

Has-miR-218-1-3p 5'- GTCGTATCCAGTGCGTGTCGTGGAGTCGGC

AATTGCACTGGATACGACCCATGG -'3

Hsa-miR-34a-5p 5'- GTCGTATCCAGTGCGTGTCGTGGAGTCGGC

AATTGCACTGGATACGACACAACC -'3

| **MicroRNA name Reverse transcription primer sequences** |
| --- |
|  |

**Supplementary Table 4. List of MicroRNA qRT-PCR Primer Sequences**

| **MicroRNA name qRT-PCR primer sequences** |
| --- |
| Hsa-miR-502-5p 5'- GGGATCCTTGCTATCTGG-'3  Has-miR-218-1-3p 5'-GGGATGGTTCCGTCAAGCA-'3  Hsa-miR-34a-5p 5'-GGGTGGCAGTGTCTTAGCT-'3  Universal reversed primers 5'- CAGTGCGTGTCGTGGAGT-'3 |
